# Disease Spectrum of Breast Cancer Susceptibility Genes

**DOI:** 10.3389/fonc.2021.663419

**Published:** 2021-04-20

**Authors:** Jin Wang, Preeti Singh, Kanhua Yin, Jingan Zhou, Yujia Bao, Menghua Wu, Kush Pathak, Sophia K. McKinley, Danielle Braun, Kevin S. Hughes

**Affiliations:** ^1^ Department of Breast Oncology, Sun Yat-sen University Cancer Center, State Key Laboratory of Oncology in South China, Collaborative Innovation Center of Cancer Medicine, Guangzhou, China; ^2^ Division of Surgical Oncology, Massachusetts General Hospital and Harvard Medical School, Boston, MA, United States; ^3^ Department of Data Sciences, Dana-Farber Cancer Institute, Boston, MA, United States; ^4^ Department of General Surgery, Beijing Anzhen Hospital, Capital Medical University, Beijing, China; ^5^ Computer Science & Artificial Intelligence, Massachusetts Institute of Technology, Boston, MA, United States; ^6^ Department of Surgical Oncology, P. D Hinduja Hospital, Mumbai, India; ^7^ Department of Surgery, Massachusetts General Hospital, Boston, MA, United States; ^8^ Department of Biostatistics, Harvard University T.H. Chan School of Public Health, Boston, MA, United States

**Keywords:** breast cancer, cancer susceptibility genes, disease spectrum, germline mutation, cancer genetic

## Abstract

**Background:**

Pathogenic variants in cancer susceptibility genes can increase the risk of a spectrum of diseases, which clinicians must manage for their patients. We evaluated the disease spectrum of breast cancer susceptibility genes (BCSGs) with the aim of developing a comprehensive resource of gene-disease associations for clinicians.

**Methods:**

Twelve genes (*ATM, BARD1, BRCA1, BRCA2, CDH1, CHEK2, NF1, PALB2, PTEN, RECQL, STK11*, and *TP53*), all of which have been conclusively established as BCSGs by the Clinical Genome Resource (ClinGen) and/or the NCCN guidelines, were investigated. The potential gene-disease associations for these 12 genes were verified and evaluated based on six genetic resources (ClinGen, NCCN, OMIM, Genetics Home Reference, GeneCards, and Gene-NCBI) and an additional literature review using a semiautomated natural language processing (NLP) abstract classification procedure.

**Results:**

Forty-two diseases were found to be associated with one or more of the 12 BCSGs for a total of 86 gene-disease associations, of which 90% (78/86) were verified by ClinGen and/or NCCN. Four gene-disease associations could not be verified by either ClinGen or NCCN but were verified by at least three of the other four genetic resources. Four gene-disease associations were verified by the NLP procedure alone.

**Conclusion:**

This study is unique in that it systematically investigates the reported disease spectrum of BCSGs by surveying multiple genetic resources and the literature with the aim of developing a single consolidated, comprehensive resource for clinicians. This innovative approach provides a general guide for evaluating gene-disease associations for BCSGs, potentially improving the clinical management of at-risk individuals.

## Introduction

Hereditary predisposition is found in approximately 10% of all breast cancer cases (1). Most are related to germline mutations in high-penetrance genes such as *BRCA1* and *BRCA2* (2–5). Since the identification of *BRCA1* and *BRCA2* (6, 7), genetic testing has become a routine part of clinical care for individuals with possible hereditary breast cancer predisposition (1). With the substantial increase in knowledge of cancer genetics (8, 9), more than 30 potential breast cancer susceptibility genes (BCSGs) have been suggested, including genes with high (e.g., *BRCA1/2, TP53, CDH1, PTEN*, and *STK11*), moderate (e.g., *PALB2, CHEK2, ATM*, and *RECQL*), and low-to-disputed penetrance (e.g., *MLH1, MSH2, MSH6, PMS2, MEN1*, and *PPM1D*) (9–12). Among them, 12 genes with high or moderate penetrance for breast cancer have been definitively established by either the Clinical Genome Resource (ClinGen) (11) or the National Comprehensive Cancer Network (NCCN) (12), the top two authoritative resources.

Pathogenic variants in a BCSG can also increase the risk of other diseases. For instance, *CDH1* is not only associated with increased breast cancer risk, but also a predisposition to gastric cancer (13, 14). Furthermore, several BCSGs are responsible for rare hereditary cancer syndromes, such as *TP53*, which is responsible for Li-Fraumeni syndrome. Individuals with this syndrome have a very high risk of developing multiple malignancies, including but not limited to, breast cancer, sarcoma, brain cancer, leukemia, lung cancer, and adrenocortical cancer (15–18). As comprehensive panel genetic testing becomes the norm (19), clinicians are increasingly faced with the challenge of advising mutation carriers about genes they may be less familiar with or involving cancer susceptibility in organs outside their specialty.

A variety of existing resources, in addition to NCCN and ClinGen, describe the diseases associated with each gene (20), including but not limited to, Genetics Home Reference (https://ghr.nlm.nih.gov/), Online Mendelian Inheritance in Man (OMIM) (https://www.ncbi.nlm.nih.gov/omim), GeneCards (https://www.genecards.org/), and Gene-NCBI (https://www.ncbi.nlm.nih.gov/gene/). However, gene-disease associations described among these six resources are often ambiguous, incomplete, or confusing. For example, the association of *BRCA2* with melanoma is identified in NCCN and Genetics Home Reference but not in other genetic resources such as ClinGen, OMIM, GeneCards, or Gene-NCBI. Furthermore, some gene-disease associations are not found in any genetic resource, such as the association of *CHEK2* with gastric cancer, which has been established with high likelihood in the literature (21, 22). This poses a considerable dilemma for clinicians who are obligated to identify and assess gene-disease associations that require management in clinical practice.

In addition, the rapidly growing medical literature makes it not possible for clinicians to extract useful information precisely and quickly. To address this challenge, Natural language processing (NLP), a technology that trains a computational algorithm with many annotated examples to allow the computer to “learn” and “predict” the meaning of human language, may present a promising solution. Our previous studies illustrate how to train and evaluate an NLP algorithm and incorporate it into a semi-automated procedure to accurately identify the penetrance studies based on abstracts (23–25).

Relying on a patchwork of resources is cumbersome, time-consuming, and can lead to errors of omission. A single comprehensive resource is critically needed to streamline this process. In light of these issues, we have developed a novel approach to identify, evaluate, and curate the diseases or complex syndromes associated with cancer susceptibility genes based on six genetic resources and the NLP literature review.

## Methods

### Established Breast Cancer Susceptibility Genes

**Figure 1:**
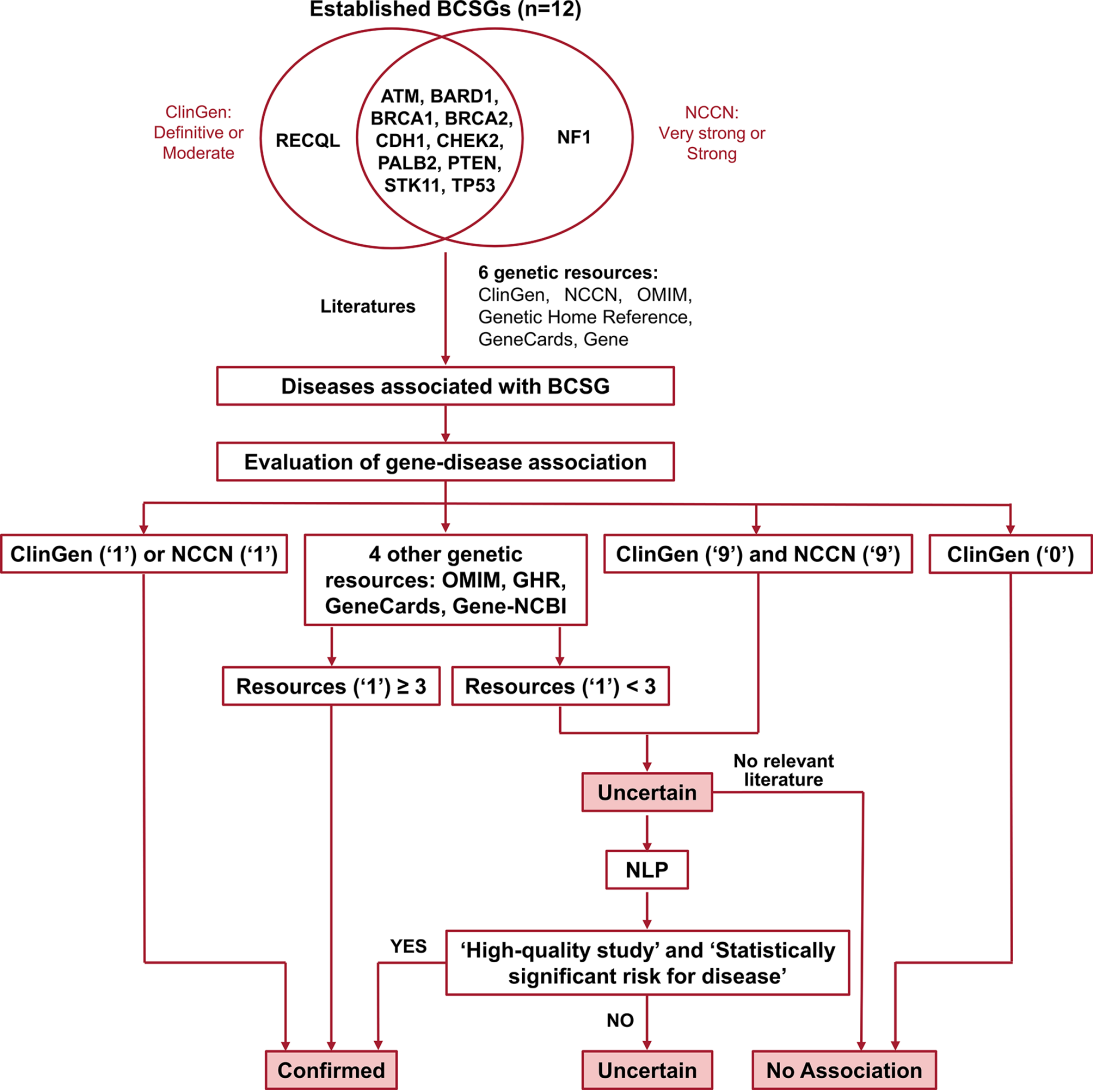
Flow chart for identifying and evaluating gene-disease association. The number ‘1’ indicates that the gene was associated with BCSG in the resource. The number ‘0’ indicates that the gene’s association with BCSG was refuted in the resource. The number ‘9’ indicates that the gene’s association with BCSG was unclear in the resource. Uncertain association indicates that the gene’s association with BCSG is unclear, and further studies are required to refute or accept the association. BCSGs, breast cancer susceptibility genes; NLP, natural language processing.

Germline genetic testing is performed on non-cancer cells and mostly blood-based or saliva-based, and a germline pathogenic variant in a cancer susceptibility gene indicates the possibility that other family members have a hereditary susceptibility to developing cancer. In contrast, somatic testing is performed on cancer cells (e.g., tumor tissue), and a somatic variant may guide targeted therapy and other treatment decisions. The present study focused on germline BCSGs, and only monoallelic BCSGs were included. The BCSGs were initially identified using ClinGen (11) and NCCN (12). In 2019, Lee and other experts on the ClinGen Hereditary Cancer Clinical Domain Executive Committee published a list of 31 high-priority genes for curation using the ClinGen Gene Curation framework (11). Among these 31 genes, 11 classified as having a ‘Definitive’ or ‘Moderate’ association with breast cancer were included in our study. The NCCN Guidelines for ‘Genetic/Familial High-Risk Assessment: Breast and Ovarian’ identified 21 genes offered in multi-gene panels where breast cancer risk was classified as ‘Very strong’, ‘Strong’, or ‘Limited’ (12). Of these 21, the 12 genes that were classified as ‘Very strong’ or ‘Strong’ were also included in our study. Accounting for overlap between the two resources, 12 BCSGs were selected for breast cancer, namely*, ATM, BARD1, BRCA1, BRCA2, CDH1, CHEK2, NF1, PALB2, PTEN, RECQL, STK11*, and *TP53* (**Figure 1**).

### Identification of Gene-Disease Association

Diseases associated with BCSGs were initially identified in the six genetic resources (ClinGen, NCCN, OMIM, Genetics Home Reference, GeneCards, and Gene-NCBI) and by reviewing the literature. For each of these sources, each potential association was coded in our database as ‘1’ if the association was definitive, ‘9’ if the association was possible, and ‘0’ if there was no association, as shown in **Supplementary Table 1**. The date of last access to all resources was November 20, 2020. In the following sections we describe in detail each of these resources.

#### ClinGen

ClinGen is a database curated by the Clinical Genome Resource. It uses a standardized clinical validity framework to assess evidence to validate a gene-disease association and to define disease management. We extracted data regarding gene-disease associations directly from the ‘Gene-Disease Validity’ reports in ClinGen (https://search.clinicalgenome.org/kb/gene-validity).

The strength of ‘Gene-Disease Validity’ was classified by ClinGen as ‘Definitive’, ‘Strong’, ‘Moderate’, ‘Limited’, ‘Refuted’, ‘Disputed’, or ‘No Reported Evidence’ based on the level of evidence. If an association was classified as ‘Definitive’, ‘Strong’, or ‘Moderate’, it was coded in our database as ‘1’ in the field ClinGen Validity. If an association was classified as ‘Limited’, it was coded in our database as ‘9’. If an association was classified as ‘Refuted’, ‘Disputed’ or ‘No Reported Evidence’, it was coded in our database as ‘0’.

We also reviewed the ‘Actionability’ reports in ClinGen, where the gene-disease associations were identified indirectly (https://clinicalgenome.org/working-groups/actionability/). The ‘Actionability’ report in ClinGen summarizes secondary findings in patients and identifies diseases caused by susceptibility genes that can be prevented or palliated. A gene-disease association was coded as ‘1’ in our database in the field ClinGen Actionability, if the disease was a manifestation of the genetic disorder, if management of that disease was recommended by screening or preventive intervention, or if the disease was verified in the ‘Penetrance’ section of the ‘Actionability’ report. The gene-disease association was coded in our database as ‘9’, if the report suggested a possible relationship.

#### NCCN Guidelines

Data was extracted from the NCCN Guidelines on Genetic/Familial High-Risk Assessment: Breast, Ovarian and Pancreatic (Version 2.2021) (12) and Colorectal (Version 2.2019) (26). A gene-disease association was coded as ‘1’ in our database if a disease or a feature was used to identify patients for genetic testing or if the management of a disease was recommended for mutation carriers. If NCCN identified a possible relationship, the gene-disease association was coded as ‘9’.

#### Other Genetic Resources

Other reputable databases such as ‘OMIM’, ‘Genetics Home Reference’, ‘GeneCards’, and ‘Gene-NCBI’ (described in detail below) were also used to identify gene-disease associations. If a gene-disease association was present in one of these resources, this association was coded as ‘1’ in our database.

‘OMIM’ is an online compendium of human genes and genetic phenotypes that is written and regularly updated by the McKusick-Nathans Institute of Genetic Medicine. The “Clinical Synopses” table for each gene was used to identify gene-disease associations.

‘Genetics Home Reference’ is a free online resource that was created after the announcement of the human genome map in 2003 and is maintained by the National Library of Medicine. It is designed to make the connection between genetics and disease more transparent for the general public. The “health conditions related to the Genetic Changes” section for each gene was used to identify gene-disease associations. Of note, as of October 1, 2020, Genetics Home Reference was ended as a stand-alone website, and most of its content has been transferred to MedlinePlus Genetics (https://medlineplus.gov/genetics).

‘GeneCards’ is a comprehensive database of human genes. The content of this database is reviewed and updated by the GeneCards Suite Project Team. The “disorders” table for each gene was used to identify gene-disease associations.

‘Gene-NCBI’ is a resource of the National Center for Biotechnology Information (NCBI), which centralizes gene-related information into individual records. Many different types of gene-specific data are connected to the record including gene products and their attributes, expression, interactions, pathways, variation, and phenotypic consequences. The “Phenotypes” section for each gene was used to identify gene-disease associations.

### Evaluation of Gene-Disease Association

The process of validating the gene-disease association is outlined in **Figure 1**. Of the six genetic resources, we considered ClinGen and NCCN the most authoritative and curated these as major resources. As shown in **Figure 1**, we designated the gene-disease association ‘verified’ if it was coded as ‘1’ in either ClinGen or NCCN. Additionally, if the gene-disease association was coded as ‘1’ in more than three other genetic resources (OMIM, Genetic Home Reference, GeneCard, and Gene-NCBI), it was also designated ‘verified’. On the other hand, we designated the gene-disease association ‘uncertain’, if it was not coded as ‘1’ in either ClinGen or NCCN and was found in fewer than three of the other genetic resources (OMIM, Genetic Home Reference, GeneCard, and Gene-NCBI). We designated the gene-disease association as ‘no association’ directly if it was coded as ‘0’ in ClinGen.

All ‘uncertain’ gene-disease associations were further evaluated by literature review using an abstract classifier NLP procedure, which classifies abstracts as being relevant to cancer penetrance or not (23, 24). Our NLP abstract classifier was developed to cull germline penetrance papers from PubMed. In brief, it uses a Support Vector Machine algorithm to classify abstracts as relevant to penetrance, prevalence, both, or neither (24). This NLP abstract classifier has been incorporated into a semiautomated procedure. The sensitivity and specificity of this approach in identifying cancer penetrance studies have been validated (23).

In this study, we used standard gene and disease PubMed search terms (**Supplementary Table 2**) to run the procedure. The NLP abstract classifier was applied to identify the abstracts that were classified as relevant to prevalence or penetrance, and the abstracts were subsequently reviewed by two researchers independently. We then retrieved the full text of these penetrance studies and determined the gene-disease associations based on the quality of the penetrance study (including type of study, sample size, carrier numbers, and ascertainment criteria) as well as the statistical significance of the results.

If no relevant penetrance abstract was identified, the association was designated ‘no association’. If relevant penetrance studies were identified, they were presented in a group consensus meeting with our principal investigator (KSH), one surgery resident, and four clinical researchers participating (two attending surgical oncologists and two research fellows in surgical oncology). The attendees selected high-quality penetrance studies based on study design, patient population, number of pathogenic variant carriers, and ascertainment mechanism, and reached a final consensus based on evaluating these high-quality studies. As a rule of thumb, we considered a gene-cancer association to be real if at least one high-quality penetrance study reported at least a two-fold increased risk that was statistically significant. If the attendees could not reach a consensus, the gene-disease association remained ‘uncertain’. Of note, to ensure accuracy, the group meeting not only discussed the potential controversial gene-cancer associations but also examined all the evidence regarding every gene-cancer association reported in the study.

## Results

### Breast Cancer Susceptibility Genes in Six Genetic Resources

As shown in **Table 1**, among the twelve established BCSGs, the association of breast cancer risk with *ATM*, *BARD1*, *BRCA1*, *BRCA2*, *CDH1*, and *CHEK2* was identified in all six genetic sources; *PALB2*, *PTEN*, *STK11* and *TP53* were identified in at least two genetic sources. However, the association of breast cancer risk with *NF1* was only identified in NCCN, and *RECQL* was only identified in ClinGen.

**Table 1:** Associations between the 12 susceptibility genes and breast cancer in six genetic resources.

Gene	Genetic Resources						
	No. of resources	ClinGen	NCCN	OMIM	GHR	GeneCards	Gene-NCBI
*ATM*	6	Definitive	Strong	1	1	1	1
*BARD1*	6	Definitive	Strong for triple-negative disease	1	1	1	1
*BRCA1*	6	Definitive	Very strong	1	1	1	1
*BRCA2*	6	Definitive	Very strong	1	1	1	1
*CDH1*	6	Definitive	Strong	1	1	1	1
*CHEK2*	6	Definitive	Strong	1	1	1	1
*STK11*	4	Definitive	Strong	1	1		
*PALB2*	4	Definitive	Strong	1		1	
*TP53*	4	Definitive	Strong		1		1
*PTEN*	3	Definitive	Strong		1		
*NF1*	1		Strong				
*RECQL*	1	Moderate					

The number ‘1’ indicates that the gene was associated with breast cancer in the resource.

GHR, Genetics Home Reference; NCBI, National Center for Biotechnology Information.

### Diseases Associated With BCSGs

There were 66 unique diseases initially identified, of which 42 diseases were determined to be associated with BCSGs by our evaluation (**Supplementary Table 3**). Besides breast cancer, malignant diseases including prostate cancer, pancreatic cancer, colorectal cancer, brain tumor, gastric cancer, ovarian cancer, and sarcoma were associated with at least three BCSGs (range: 3 to 6). However, *BARD1* and *RECQL* were only associated with breast cancer, without increased risk for any other diseases.

The disease spectrum of each BCSG is shown in **Table 2**. Furthermore, several BCSGs are associated with specific syndromes, such as *NF1* with Neurofibromatosis Type 1, *PTEN* with Cowden Syndrome, *STK11* with Peutz-Jeghers Syndrome, and *TP53* with Li-Fraumeni Syndrome. The most common cancers associated with these syndromes were determined to be associated with the corresponding susceptibility genes by our procedure.

**Table 2:** Diseases associated with the 12 breast cancer susceptibility genes.

BCSGs	Disease Spectrum		
	Malignant	Benign	Borderline
*ATM*	Breast Cancer, Colorectal Cancer, Gastric Cancer, Pancreatic Cancer, Prostate Cancer		
*BARD1*	Breast Cancer		
*BRCA1*	Breast Cancer, Ovarian Cancer, Pancreatic Cancer, Prostate Cancer		
*BRCA2*	Breast Cancer, Melanoma, Ovarian Cancer, Pancreatic Cancer, Prostate Cancer		
*CDH1*	Breast Cancer, Gastric Cancer	BCD Syndrome*	
*CHEK2*	Breast Cancer, Colorectal Cancer, Gastric Cancer, Kidney Cancer, Prostate Cancer, Osteosarcoma, Thyroid Cancer		
*NF1*	Brain Tumor, Breast Cancer, Leukemia, Sarcoma	Bone Dysplasia, Cafe-Au-Lait Spots, Intellectual Disability, Iris Hamartoma, Neurofibroma, Pulmonary Stenosis, Skin	GIST, Paraganglioma, Pheochromocytoma
*PALB2*	Breast Cancer, Ovarian Cancer, Pancreatic Cancer, Prostate Cancer		
*PTEN*	Brain Tumor, Breast Cancer, Colorectal Cancer, Endometrial Cancer, Kidney Cancer, Melanoma, Thyroid Cancer	Acral Keratoses, Autism, Cerebrovascular Malformation, Facial Papules, GI Hamartomatous Polyps, Lipoma, Macrocephaly, Macular Pigmentation, Oral Mucosal Papillomatosis, Palmoplantar Keratoses, Thyroid, Trichilemmoma, Uterine Fibroid	
*RECQL*	Breast Cancer		
*STK11*	Breast Cancer, Cervical Cancer, Colorectal Cancer, Endometrial Cancer, Gastric Cancer, Hepatobiliary Cancer, Lung Cancer, Pancreatic Cancer, Small Intestine Cancer	GI Hamartomatous Polyps, Skin	Non-Epithelial Ovarian Tumor, Ovarian SCST, Testicular SCST
*TP53*	Adrenocortical Carcinoma, Brain Tumor, Breast Cancer, Colorectal Cancer, Hepatobiliary Cancer, Pancreatic Cancer, Osteosarcoma, Soft Tissue Sarcoma		

GI, gastrointestinal; BCD, blepharocheilodontic; SCST, sex cord-stromal tumor; GIST, gastrointestinal stromal tumor.

*BCD syndrome consists of facial dysmorphism, hypertelorism, imperforate anus, distichiasis, clinodactyly, hypoplastic nails, choanal atresia, cleft palate, and benign teeth disorder.

### Disease Spectrum of BCSGs and the Corresponding Resources

**Figure 2:**
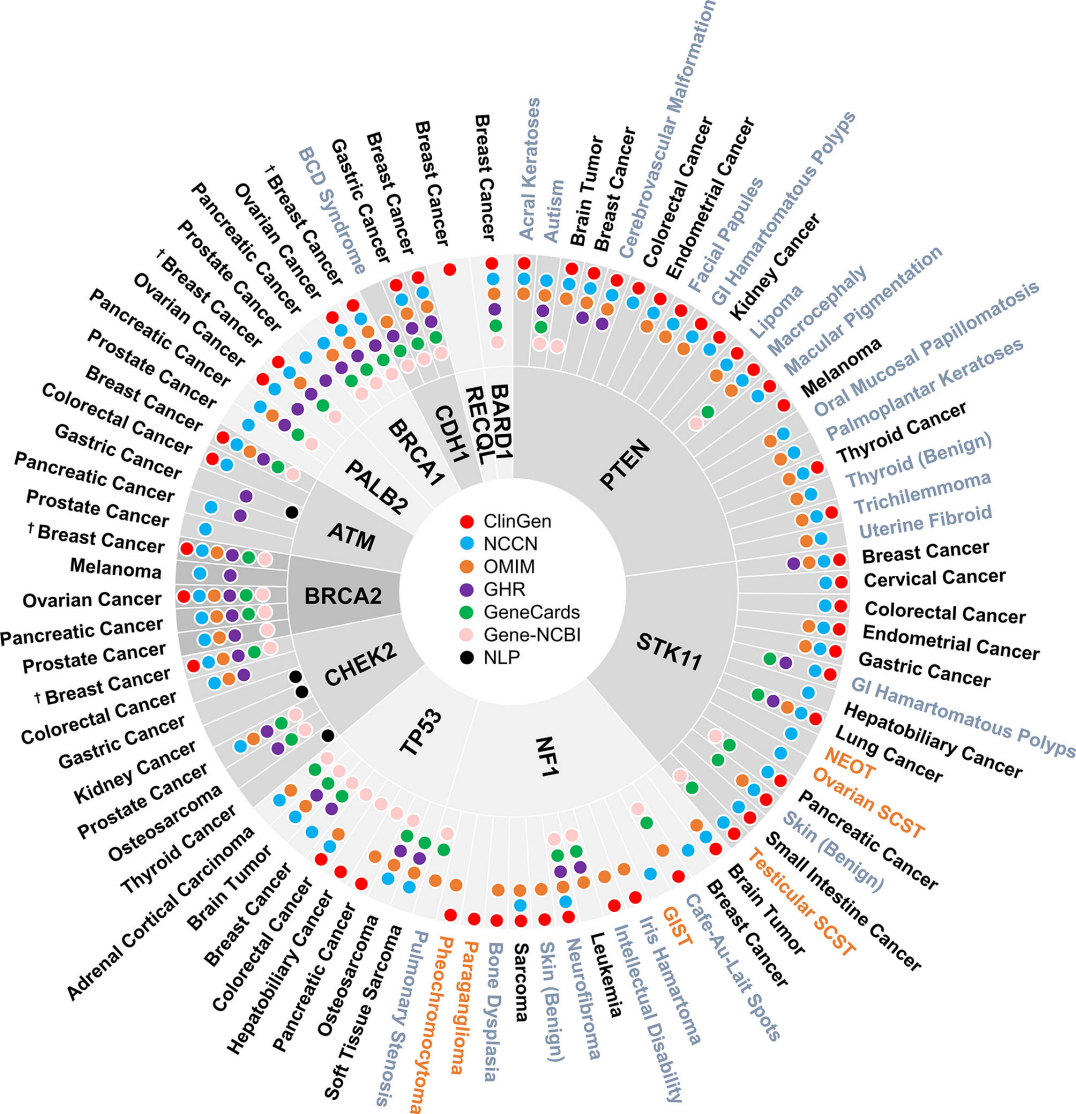
Disease spectrum of breast cancer susceptibility genes. “†” refers to both female and male breast cancer. The three colors represent malignant disease (black), benign disease (grey), and borderline disease (orange), respectively. NLP, natural language processing; GI, gastrointestinal; BCD, blepharocheilodontic syndrome; SCST, sex cord-stromal tumor; GIST, gastrointestinal stromal tumor; NEOT, non-epithelial ovarian tumor.

A total of 160 gene-disease associations were initially identified in the six genetic resources and literature (**Supplementary Table 1**). As shown in **Figure 2**, a total of 86 gene-disease associations were identified by our evaluation. Among them, 90% (78/86) of gene-disease associations were verified by ClinGen and/or NCCN. Conversely, four gene-disease associations were absent from both ClinGen and NCCN but verified in three or more of the other four genetic resources. These included *CDH1*-Blepharocheilodontic (BCD) Syndrome, *CHEK2*-osteosarcoma, *NF1*-leukemia, and *NF1*-pulmonary stenosis. Notably, four gene-disease associations, namely, *ATM*-gastric cancer, *CHEK2*-gastric cancer, *CHEK2*-kidney cancer, and *CHEK2*-thyroid cancer, were verified by NLP literature review alone.

## Discussion

Although hereditary breast cancer is mainly associated with *BRCA1/2* pathogenic variants, it may also be associated with germline mutations in other genes. Thus, multi-gene panels usually include both high- and moderate-penetrance genes associated with breast cancer (8, 27, 28). The twelve BCSGs included in our study are those previously established by ClinGen and/or NCCN. To outline the disease spectrum for the twelve BCSGs, we examined six reliable genetic resources combined with a literature review using NLP. Finally, 49 unique diseases were verified as being associated with the twelve BCSGs.

One of the authoritative resources used for this study is the NIH-funded ClinGen. In contrast to “expert panel” consensus assessments used by NCCN, ClinGen creates a framework that provides evidence for the strength of the association between a gene and a disease risk through semi-quantitative classification (29). The ClinGen classification is based on genetic evidence including case-level data and case-control data, as well as experimental evidence. The other authoritative resource employed for this study is the NCCN Guidelines - the recognized standard for clinical practice in cancer care - using its frequently updated set of clinical practice guidelines. More than 1,300 physicians and oncology researchers from the NCCN Member Institutions comprise the expert panels. Hence, the gene-disease association was designated ‘verified’ in our study if it was established by either ClinGen or NCCN. Although the standardized literature review method used by ClinGen is outstanding (11), this approach is time-consuming and leads to delay in reflecting the most recent findings. In addition, the gene-cancer associations listed on the NCCN guidelines may not be comprehensive. Therefore, it is necessary to include other genetic resources and find associations missed or not yet addressed by ClinGen and/or NCCN.

Four other genetic resources (OMIM, Genetics Home Reference, GeneCards, and Gene-NCBI) are also considered reputable and contain a comprehensive compendium of relationships between phenotypes and genotypes. However, these resources lack the strict curation processes for evaluating strength of evidence utilized by ClinGen or the expert panels employed by NCCN. Therefore, we rated the level of evidence from these four resources lower than ClinGen and NCCN, and the gene-disease association was designated ‘verified’ only if it was established by at least three of these sources when the relationship was not found in ClinGen or NCCN. Meanwhile, we understand that the likely valid gene-disease associations we identified that were not present in ClinGen or NCCN may be explained in part by the observation that the latter entities work in a slow and deliberate manner that might not yet have allowed a full review of all associations.

Forty-nine unique diseases were verified as being associated with BCSGs by our procedure. Each BCSG was associated with at least three diseases except *BARD1* and *RECQL*, which were only associated with breast cancer. *BARD1* shares strong structural homology with *BRCA1* and has been demonstrated to be involved in the cellular DNA repair process (30). The association between breast cancer and mutations in the *BARD1* gene was first found in a large case-control study of 65,057 women with breast cancer (8), where the prevalence of *BARD1* mutations was 0.18%, significantly greater than the controls (OR = 2.16, 95% CI: 1.31-3.63, *p* < 0.05). On the other hand, *RECQL* was first identified as a novel breast cancer susceptibility gene in 2015, by two independent research groups (31, 32). Bogdanova et al. compared 2596 breast cancer patients and 2132 healthy females from central Europe and indicated that RECQL* c.1667_1667+3delAGTA could represent a moderate-risk breast cancer susceptibility allele (33). A recent study found a moderate risk of breast cancer in African American women with *RECQL* mutation (34). In addition, *RECQL* is considered associated with hereditary breast carcinoma in ClinGen (gene-disease validity: moderate) (https://search.clinicalgenome.org/kb/genes/HGNC:9948). However, there is no high-quality penetrance study that showed statistical significance for additional diseases beyond breast cancer.

Generally speaking the BCSGs are thought to affect female breast cancer risk, but some are also associated with male breast cancer (MBC). Tai et al. evaluated 97 men with breast cancer from 1939 families. The cumulative risk of breast cancer was higher in both *BRCA1* and *BRCA2* male heterozygotes compared to those without a *BRCA1/2* pathogenic variant at all ages. The relative risk of developing breast cancer peaks in the 30s and 40s (35). Another study analyzed 321 families with *BRCA2* mutations both retrospectively and prospectively, suggesting a cumulative risk for male breast cancer of 8.9% up to age 80 (36). Based on these data, NCCN guidelines recommend that men with a *BRCA1/2* pathogenic variant should receive a clinical breast exam at a young age (12).

Notably, we found that *CHEK2* and *PALB2* were also associated with male breast cancer in GeneCards. We verified these associations by literature review based on the NLP procedure, with the literature showing strong evidence in penetrance studies. The *CHEK2/1100delC*, a truncating variant, is present in 13.5% of individuals from families with male breast cancer (*p* = 0.00015) and results in an approximately ten-fold increase of breast cancer risk in men (37). A population-based study found the *CHEK2/1100delC* was present in 4.2% of unselected male breast cancer cases, more prevalent than the frequency of 1.1% in 1,692 controls (OR = 4.1, 95% CI: 1.2-14.3, *p* = 0.05) (38). Recently, Yang et al. analyzed data from 524 families with *PALB2* pathogenic variants from 21 countries and found an association between *PALB2* and risk of male breast cancer (RR = 7.34, 95% CI: 1.28-42.18, *p* = 0.026) (39). Additionally, Pritzlaff et al. reviewed 715 male breast cancer patients who underwent germline multi-gene panel testing and found that pathogenic variants in *CHEK2* (OR = 3.7, *p* = 6.24 × 10^-24^) and *PALB2* (OR = 6.6, *p* = 0.01) were both significantly associated with breast cancer risk in men (40).

In the present study, 82% of gene-disease associations were verified by ClinGen and/or NCCN, underscoring the credibility of these two major resources. Nevertheless, six gene-disease associations were not found in ClinGen or NCCN but were instead identified in at least three of the other four genetic resources. Furthermore, these associations were similarly supported by published studies with strong evidence of the association, underscoring the reliability our review criteria.

Of note, four gene-disease associations, i.e., *ATM*-gastric cancer, *CHEK2*-gastric cancer, *CHEK2*-kidney cancer, and *CHEK2*-thyroid cancer, were not identified in any of the six resources but were verified by the NLP-aided literature review. In 2015, Helgason et al. reported a GWAS of gastric cancer in a European population, using information on 2,500 population-based gastric cancer cases and 205,652 controls. They found a new gastric cancer association with loss-of-function mutations in *ATM* (OR = 4.74, *p* = 8.0 × 10^-12^) (41). A recent study reported that *ATM* carriers were significantly associated with lower protein expression in five cancer types, including gastric cancer (42). A *CHEK2* mutation was also identified to predispose to gastric cancer (OR = 1.6, *p* = 0.004), particularly in young-onset cases (OR = 2.1, *p* = 0.01) (21). Additionally, Näslund-Koch et al. examined 86,975 individuals from the Copenhagen General Population Study. The age- and sex-adjusted hazard ratio for *CHEK2/1100delC* heterozygotes compared with noncarriers was 5.76 (95% CI: 2.12-15.6) for gastric cancer and 3.61 (95% CI: 1.33-9.79) for kidney cancer (22). Furthermore, a case-control study reported a *CHEK2* mutation in 15.6% of unselected patients with papillary thyroid cancer, compared to 6.0% in age- and sex-matched controls (OR = 3.3, *p* < 0.0001) (43). Another *CHEK2* variant, c.470C allele, was shown to increase the risk of papillary thyroid carcinoma in female patients by almost 13-fold (OR = 12.81, *p* = 0.019) (44).

The NCCN guidelines for considering risk-reducing mastectomy and breast MRI are well established for carriers of high-risk genes (e.g., *BRCA1, BRCA2*, and *PALB2*), and guidelines on annual mammogram with consideration of breast MRI are also established regarding carriers with moderate-risk genes (e.g., *ATM* and *CHEK2*) (12). Women with genes such as *TP53, CDH1, PTEN, STK11*, and *NF1* may be managed according to established guidelines for the associated cancer predisposition syndrome. For instance, in Li-Fraumeni syndrome, annual whole-body MRI is advised in *TP53* pathogenic variant carriers (45, 46). More aggressive interventions may be recommended, such as consideration of prophylactic gastrectomy if a *CDH1* mutation is found, even in the absence of gastric cancer in the family (47). This necessitates that clinicians stay current with management guidelines and access reliable information resources to implement these updates effectively for their patients (e.g., resources such as ASK2ME could aid with this). Risks of other cancers for those BCSG carriers appear to be modestly elevated, but whether this should alter screening recommendations is unknown. For example, the risk of leukemia with “*TP53*” is 1.6 times as high as the general population, but since the general population risk of leukemia is 0.9%, this amounts to an absolute risk of only 1.4% by age 85 (48). Although a pathogenic mutation in *TP53* is statistically associated with leukemia, it would be hard to justify intensive screening or prevention measures based on this information. It is beyond the scope of this paper to identify the penetrance for each gene-disease association, but this will be the target of future work. Our proposed expansion of disease-gene association reporting will require clinicians to counsel patients appropriately about their risk of additional diseases and to refer them to genetic counselors or other specialists (e.g., neurologist, urologist).

Evaluation based on six genetic resources could result in omissions of some phenotypes associated with BCSGs. We attempted to lessen this effect by including a literature review as an additional step. Another limitation is that the strict criteria we set for gene-disease associations (e.g., verified by ClinGen/NCCN, or at least three genetic resources) could mean that some diseases are overlooked. By reviewing the literature using NLP, we reevaluated those uncertain gene-disease associations to lessen this effect as much as possible. Although the comprehensiveness of our data seems to be conducive to more individualized care, this raises the problem of absence of management guidelines for patients who carry such variants. Additionally, the clinical utility of identifying potential diseases in BCSG carriers may conflict with current cost-efficacy constraints (i.e., interpreting variants, genetic counseling, overdiagnoses, and resulting anxiety in patients). Of note, we are making assumptions based on the available evidence, and we recognize that authoritative sources, such as ClinGen and NCCN guidelines, are updated periodically. Thus, this study represents a snapshot of current knowledge and understanding, rather than a definitive conclusion.

In 2016, we built a clinical decision support tool for cancer susceptibility genes, called Ask2Me.Org (49). This tool provides labs, researchers, and clinical experts with the estimated cancer risk of germline pathogenic variants, including the disease spectrum for each susceptibility gene. Ask2Me.Org has been recommended as a resource in recent clinical practice guidelines (50). These disease spectrums we verified in the current study will be soon available in our website Ask2Me.Org, which is constantly updated. Ongoing research based on accurate estimates of cancer risk needs to be conducted in terms of appropriate management strategies.

## Conclusions

To the best of our knowledge, this is the first study to collate the disease spectrum of BCSGs from multiple sources and make it available in a single resource. Notably, we developed an innovative assessment process based on six genetic resources and literature review using an NLP procedure. Throughout our evaluation process, we have kept in mind that frequent updates of the disease spectrum will be necessary to adjust for new data in these genetic resources. Our study provides a reference point for future studies, showing that BCSG mutation carriers should also be cautious of other diseases beyond breast cancer and highlights the necessity of broadening the criteria of management and improving outcomes for at-risk individuals.

## Data Availability Statement

The raw data supporting the conclusions of this article will be made available by the authors, without undue reservation.

## Ethics Statement

We used public database with no patient data, and individual informed consent was waived.

## Author Contributions

JW, KY, DB, and KSH were involved in the conceptualization and design of this study. JW, PS, KY, JZ, KP, and SKM collected the data. YB and MW were responsible for maintaining the natural language processing abstract classifier. JW and PS analyzed the data and interpreted the results. JW, PS, and KY drafted the initial manuscript with critical feedback from DB and KSH. All authors contributed to the article and approved the submitted version.

## Conflict of Interest

KH receives Honoraria from Hologic (Surgical implant for radiation planning with breast conservation and wire-free breast biopsy) and Myriad Genetics and has a financial interest in CRA Health (Formerly Hughes RiskApps). CRA Health develops risk assessment models/software with a particular focus on breast cancer and colorectal cancer. KH is a founder and owns equity in the company. KH is the Co-Creator of Ask2Me.Org, which is freely available for clinical use and is licensed for commercial use by the Dana Farber Cancer Institute and the MGH. KH’s interests in CRA Health and Ask2Me.Org were reviewed and are managed by Massachusetts General Hospital and Partners Health Care in accordance with their conflict of interest policies. DB co-leads the BayesMendel laboratory, which licenses software for the computation of risk prediction models. She does not derive any personal income from these licenses. All revenues are assigned to the lab for software maintenance and upgrades.

The remaining authors declare that the research was conducted in the absence of any commercial or financial relationships that could be construed as a potential conflict of interest.

## References

[1] Collaborative Group on Hormonal Factors in Breast C. Familial breast cancer: collaborative reanalysis of individual data from 52 epidemiological studies including 58,209 women with breast cancer and 101,986 women without the disease. Lancet (2001) 358:1389–99. 10.1016/S0140-6736(01)06524-2 11705483

[2] Risch HA McLaughlin JR Cole DE Rosen B Bradley L Fan I. Population BRCA1 and BRCA2 mutation frequencies and cancer penetrances: A kin-cohort study in Ontario, Canada. J Natl Cancer Inst (2006) 98:1694–706. 10.1093/jnci/djj465 17148771

[3] Begg CB Haile RW Borg A Malone KE Concannon P Thomas DC. Variation of breast cancer risk among BRCA1/2 carriers. JAMA (2008) 299:194–201. 10.1001/jama.2007.55-a 18182601 PMC2714486

[4] Chen S Parmigiani G. Meta-analysis of BRCA1 and BRCA2 penetrance. J Clin Oncol (2007) 25:1329–33. 10.1200/JCO.2006.09.1066 PMC2267287 17416853

[5] Mavaddat N Peock S Frost D Ellis S Platte R Fineberg E. Embrace. Cancer risks for BRCA1 and BRCA2 mutation carriers: results from prospective analysis of EMBRACE. J Natl Cancer Inst (2013) 105:812–22. 10.1093/jnci/djt095 23628597

[6] Miki Y Swensen J Shattuck-Eidens D Futreal PA Harshman K Tavtigian S. A strong candidate for the breast and ovarian cancer susceptibility gene BRCA1. Science (1994) 266:66–71. 10.1126/science.7545954 7545954

[7] Wooster R Bignell G Lancaster J Swift S Seal S Mangion J. Identification of the breast cancer susceptibility gene BRCA2. Nature (1995) 378:789–92. 10.1038/378789a0 8524414

[8] Couch FJ Shimelis H Hu C Hart SN Polley EC Na J. Associations Between Cancer Predisposition Testing Panel Genes and Breast Cancer. JAMA Oncol (2017) 3:1190–6. 10.1001/jamaoncol.2017.0424 PMC5599323 28418444

[9] Easton DF Pharoah PD Antoniou AC Tischkowitz M Tavtigian SV Nathanson K. Gene-panel sequencing and the prediction of breast-cancer risk. N Engl J Med (2015) 372:2243–57. 10.1056/NEJMsr1501341 PMC4610139 26014596

[10] Kean S. Breast cancer. The ‘other’ breast cancer genes. Science (2014) 343:1457–9. 10.1126/science.343.6178.1457 24675950

[11] Lee K Seifert BA Shimelis H Ghosh R Crowley SB Carter NJ. Clinical validity assessment of genes frequently tested on hereditary breast and ovarian cancer susceptibility sequencing panels. Genet Med (2019) 21:1497–506. 10.1038/s41436-018-0361-5 PMC6579711 30504931

[12] Daly MB Pilarski R Berry MP Buys SS Dickson P Domchek SM. Genetic/Familial High-Risk Assessment: Breast, Ovarian, and Pancreatic, Version 2.2021, NCCN Clinical Practice Guidelines in Oncology. J Natl Compr Canc Netw (2021) 19(1):77–102. 10.6004/jnccn.2021.0001 33406487

[13] Pharoah PD Guilford P Caldas C. International Gastric Cancer Linkage Consortium. Incidence of gastric cancer and breast cancer in CDH1 (E-cadherin) mutation carriers from hereditary diffuse gastric cancer families. Gastroenterology (2001) 121:1348–53. 10.1053/gast.2001.29611 11729114

[14] Hansford S Kaurah P Li-Chang H Woo M Senz J Pinheiro H. Hereditary Diffuse Gastric Cancer Syndrome: CDH1 Mutations and Beyond. JAMA Oncol (2015) 1:23–32. 10.1001/jamaoncol.2014.168 26182300

[15] Malkin D. Li-fraumeni syndrome. Genes Cancer (2011) 2:475–84. 10.1177/1947601911413466 PMC3135649 21779515

[16] Schneider K Zelley K Nichols KE Garber J. Li-Fraumeni syndrome. Adam MP Ardinger HH Pagon RA, editors. GeneReviews University of Washington Seattle (1993-2021). 20301488

[17] Nichols KE Malkin D Garber JE Fraumeni JF Jr Li FP. Germ-line p53 mutations predispose to a wide spectrum of early-onset cancers. Cancer Epidemiol Biomarkers Prev (2001) 10:83–7. 11219776

[18] Gonzalez KD Noltner KA Buzin CH Gu D Wen-Fong CY Nguyen VQ. Beyond Li Fraumeni syndrome: clinical characteristics of families with p53 germline mutations. J Clin Oncol (2009) 27:1250–6. 10.1200/JCO.2008.16.6959 19204208

[19] Wideroff L Vadaparampil ST Greene MH Taplin S Olson L Freedman AN. Hereditary breast/ovarian and colorectal cancer genetics knowledge in a national sample of US physicians. J Med Genet (2005) 42:749–55. 10.1136/jmg.2004.030296 PMC1735923 15784723

[20] Rehm HL Berg JS Brooks LD Bustamante CD Evans JP Landrum MJ. ClinGen-the Clinical Genome Resource. N Engl J Med (2015) 372:2235–42. 10.1056/NEJMsr1406261 PMC4474187 26014595

[21] Teodorczyk U Cybulski C Wokołorczyk D Jakubowska A Starzyńska T Lawniczak M. The risk of gastric cancer in carriers of CHEK2 mutations. Fam Cancer (2013) 12:473–8. 10.1007/s10689-012-9599-2 23296741

[22] Näslund-Koch C Nordestgaard BG Bojesen SE. Increased Risk for Other Cancers in Addition to Breast Cancer for CHEK2*1100delC Heterozygotes Estimated From the Copenhagen General Population Study. J Clin Oncol (2016) 34:1208–16. 10.1200/JCO.2015.63.3594 26884562

[23] Deng Z Yin K Bao Y Armengol VD Wang C Tiwari A. Validation of a Semiautomated Natural Language Processing-Based Procedure for Meta-Analysis of Cancer Susceptibility Gene Penetrance. JCO Clin Cancer Inform (2019) 3:1–9. 10.1200/CCI.19.00043 PMC6873944 31419182

[24] Bao Y Deng Z Wang Y Kim H Armengol VD Acevedo F. Using Machine Learning and Natural Language Processing to Review and Classify the Medical Literature on Cancer Susceptibility Genes. JCO Clin Cancer Inform (2019) 3:1–9. 10.1200/CCI.19.00042 PMC6873946 31545655

[25] Hughes KS Zhou J Bao Y Singh P Wang J Yin K. Natural language processing to facilitate breast cancer research and management. Breast J (2020) 26:92–9. 10.1111/tbj.13718 31854067

[26] Gupta S Provenzale D Llor X Halverson AL Grady W Chung DC. NCCN Guidelines Insights: Genetic/Familial High-Risk Assessment: Colorectal, Version 2.2019. J Natl Compr Canc Netw (2019) 17:1032–41. 10.6004/jnccn.2019.0044 31487681

[27] Kurian AW Hare EE Mills MA Kingham KE McPherson L Whittemore AS. Clinical evaluation of a multiplegene sequencing panel for hereditary cancer risk assessment. J Clin Oncol (2014) 32:2001–9. 10.1200/JCO.2013.53.6607 PMC4067941 24733792

[28] Tung N Battelli C Allen B Kaldate R Bhatnagar S Bowles K. Frequency of mutations in individuals with breast cancer referred for BRCA1 and BRCA2 testing using next generation sequencing with a 25-gene panel. Cancer (2015) 121:25–33. 10.1002/cncr.29010 25186627

[29] Strande NT Riggs ER Buchanan AH Ceyhan-Birsoy O DiStefano M Dwight SS. Evaluating the Clinical Validity of Gene-Disease Associations: An Evidence-Based Framework Developed by the Clinical Genome Resource. Am J Hum Genet (2017) 100:895–906. 10.1016/j.ajhg.2017.04.015 28552198 PMC5473734

[30] Wu LC Wang ZW Tsan JT Spillman MA Phung A Xu XL. Identification of a RING protein that can interact in vivo with the BRCA1 gene product. Nat Genet (1996) 14:430–40. 10.1038/ng1296-430 8944023

[31] Cybulski C Carrot-Zhang J Kluźniak W Rivera B Kashyap A Wokołorczyk D. Germline RECQL mutations are associated with breast cancer susceptibility. Nat Genet (2015) 47:643–6. 10.1038/ng.3284 25915596

[32] Sun J Wang Y Xia Y Xu Y Ouyang T Li J. Mutations in RECQL gene are associated with predisposition to breast cancer. PloS Genet (2015) 11:e1005228. 10.1371/journal.pgen.1005228 25945795 PMC4422667

[33] Bogdanova N Pfeifer K Schürmann P Antonenkova N Siggelkow W Christiansen H. Analysis of a RECQL splicing mutation, c.1667_1667+3delAGTA, in breast cancer patients and controls from Central Europe. Fam Cancer (2017) 16(2):181–6. 10.1007/s10689-016-9944-y 27832498

[34] Palmer JR Polley EC Hu C John EM Haiman C Hart SN. Contribution of germline predisposition gene mutations to breast cancer risk in African American women. J Natl Cancer Inst (2020) 112(12):1213–21. 10.1093/jnci/djaa040 PMC7735769 32427313

[35] Tai YC Domchek S Parmigiani G Chen S. Breast cancer risk among male BRCA1 and BRCA2 mutation carriers. J Natl Cancer Inst (2007) 99:1811–4. 10.1093/jnci/djm203 PMC2267289 18042939

[36] Evans DG Susnerwala I Dawson J Woodward E Maher ER Lalloo F. Risk of breast cancer in male BRCA2 carriers. J Med Genet (2010) 47:710–1. 10.1136/jmg.2009.075176 20587410

[37] Meijers-Heijboer H van den Ouweland A Klijn J Wasielewski M de Snoo A Oldenburg R. CHEK2-Breast Cancer Consortium. Low-penetrance susceptibility to breast cancer due to CHEK2(*)1100delC in noncarriers of BRCA1 or BRCA2 mutations. Nat Genet (2002) 31:55–9. 10.1038/ng879 11967536

[38] Wasielewski M den Bakker MA van den Ouweland A Meijer-van Gelder ME Portengen H Klijn JG. CHEK2 1100delC and male breast cancer in the Netherlands. Breast Cancer Res Treat (2009) 116:397–400. 10.1007/s10549-008-0162-7 18759107

[39] Yang X Leslie G Doroszuk A Schneider S Allen J Decker B. Cancer Risks Associated with Germline PALB2 Pathogenic Variants: An International Study of 524 Families. J Clin Oncol (2020) 38:674–85. 10.1200/JCO.19.01907 PMC7049229 31841383

[40] Pritzlaff M Summerour P McFarland R Li S Reineke P Dolinsky JS. Male breast cancer in a multi-gene panel testing cohort: insights and unexpected results. Breast Cancer Res Treat (2017) 161:575–86. 10.1007/s10549-016-4085-4 PMC5241330 28008555

[41] Helgason H Rafnar T Olafsdottir HS Jonasson JG Sigurdsson A Stacey SN. Loss-of-function variants in ATM confer risk of gastric cancer. Nat Genet (2015) 47:906–10. 10.1038/ng.3342 26098866

[42] Huang KL Mashl RJ Wu Y Ritter DI Wang J Oh C. Pathogenic Germline Variants in 10,389 Adult Cancers. Cell (2018) 173:355–70.e14. 10.1016/j.cell.2018.03.039 29625052 PMC5949147

[43] Siołek M Cybulski C Gąsior-Perczak D Kowalik A Kozak-Klonowska B Kowalska A. CHEK2 mutations and the risk of papillary thyroid cancer. Int J Cancer (2015) 137:548–52. 10.1002/ijc.29426 25583358

[44] Kaczmarek-Ryś M Ziemnicka K Hryhorowicz ST Górczak K Hoppe-Gołębiewska J Skrzypczak-Zielińska M. The c.470 T > C CHEK2 missense variant increases the risk of differentiated thyroid carcinoma in the Great Poland population. Hered Cancer Clin Pract (2015) 13:8. 10.1186/s13053-015-0030-5 25798211 PMC4367841

[45] Ballinger ML Best A Mai PL Khincha PP Loud JT Peters JA. Baseline Surveillance in Li-Fraumeni Syndrome Using Whole-Body Magnetic Resonance Imaging: A Meta-analysis. JAMA Oncol (2017) 3:1634–9. 10.1001/jamaoncol.2017.1968 PMC5824277 28772291

[46] Piombino C Cortesi L Lambertini M Punie K Grandi G Toss A. Secondary Prevention in Hereditary Breast and/or Ovarian Cancer Syndromes Other Than BRCA. J Oncol (2020) 2020:6384190. 10.1155/2020/6384190 32733558 PMC7376433

[47] Lynce F Isaacs C. How Far Do We Go With Genetic Evaluation? Gene Panel Tumor Testing Am Soc Clin Oncol Educ Book (2016) 35:e72–8. 10.1200/EDBK_160391 PMC6054472 27249773

[48] Mai PL Best AF Peters JA DeCastro RM Khincha PP Loud JT. Risks of first and subsequent cancers among TP53 mutation carriers in the National Cancer Institute Li-Fraumeni syndrome cohort. Cancer (2016) 122:3673–81. 10.1002/cncr.30248 PMC5115949 27496084

[49] Braun D Yang J Griffin M Parmigiani G Hughes KS. A clinical decision support tool to predict cancer risk for commonly tested cancer-related germline mutations. J Genet Couns (2018) 27:1187–99. 10.1007/s10897-018-0238-4 PMC6240422 29500626

[50] Manahan ER Kuerer HM Sebastian M Hughes KS Boughey JC Euhus DM. Consensus guidelines on genetic testing for hereditary breast cancer from the American Society of Breast Surgeons. Ann Surg Oncol (2019) 26:3025–31. 10.1245/s10434-019-07549-8 PMC6733830 31342359

