# Supplementary material for: Disease Spectrum of Breast Cancer Susceptibility Genes
Source: Front Oncol. 2021 Apr 20;11:663419. doi: 10.3389/fonc.2021.663419 (PMC8093501; doi:10.3389/fonc.2021.663419)
Supplement: Supplementary file 1 [file Table_1.docx]

**Supplementary Table 1. Initial data of gene-disease association with code in six genetic resources**

| **Genes** | **Diseases** | **Predominant Subtype** | **Association** | **ClinGen** | **NCCN** | **OMIM** | **GHR** | **Gene**  **Cards** | **Gene-**  **NCBI** |
| --- | --- | --- | --- | --- | --- | --- | --- | --- | --- |
| *ATM* | Brain Tumor | Glioblastoma Multiforme | No Association |  |  |  |  | 1 |  |
|  | Breast Cancer |  | Verified | 1 | 1 | 1 | 1 | 1 | 1 |
|  | Colorectal Cancer |  | Verified | 1 | 1 |  |  |  |  |
|  | Gastric Cancer |  | Verified |  |  |  | 1 |  |  |
|  | Lung Cancer | NSCLC | No Association |  |  |  | 1 |  |  |
|  | Melanoma |  | No Association |  |  |  | 1 |  |  |
|  | Ovarian Cancer |  | Uncertain | 9 | 9 |  | 1 |  |  |
|  | Pancreatic Cancer |  | Verified |  | 1 |  | 1 |  |  |
|  | Prostate Cancer |  | Verified |  | 1 |  |  |  |  |
| *BARD1* | Breast Cancer |  | Verified | 1 | 1 | 1 | 1 | 1 | 1 |
|  | Colorectal Cancer |  | No Association | 9 |  |  |  |  |  |
|  | Ovarian Cancer |  | Uncertain | 9 |  |  | 1 | 1 |  |
| *BRCA1* | Breast Cancer |  | Verified | 1 | 1 | 1 | 1 | 1 | 1 |
|  | Colorectal Cancer |  | No Association |  |  |  | 1 |  |  |
|  | Gastric Cancer |  | Uncertain |  | 9 |  | 1 |  |  |
|  | Ovarian Cancer |  | Verified | 1 | 1 | 1 | 1 | 1 | 1 |
|  | Pancreatic Cancer |  | Verified |  | 1 | 1 | 1 | 1 | 1 |
|  | Prostate Cancer |  | Verified |  | 1 |  | 1 |  |  |
| *BRCA2* | Breast Cancer |  | Verified | 1 | 1 | 1 | 1 | 1 | 1 |
|  | Brain Tumor |  | No Association |  |  |  |  |  |  |
|  | Gastric Cancer |  | Uncertain |  | 9 |  |  |  |  |
|  | Melanoma |  | Verified |  | 1 |  | 1 |  |  |
|  | Ovarian Cancer |  | Verified | 1 | 1 | 1 | 1 | 1 | 1 |
|  | Pancreatic Cancer |  | Verified |  | 1 | 1 | 1 | 1 | 1 |
|  | Prostate Cancer |  | Verified |  | 1 | 1 | 1 |  | 1 |
| *CDH1* | Breast Cancer |  | Verified | 1 | 1 | 1 | 1 | 1 | 1 |
|  | Central Nervous System (Benign) | Neural tube defect | Uncertain |  |  | 1 |  |  |  |
|  | Colorectal Cancer |  | No Association | 9 |  |  |  |  |  |
|  | Endometrial Cancer |  | No Association |  |  | 1 |  |  | 1 |
|  | Eye (Benign) | Hypertelorism | Verified |  |  | 1 | 1 |  |  |
|  | Facial Dysmorphism |  | Verified |  |  | 1 |  |  |  |
|  | Gastric Cancer |  | Verified | 1 | 1 | 1 | 1 | 1 | 1 |
|  | Gastrointestinal (Benign) | Imperforate anus | Verified |  |  | 1 |  |  |  |
|  | Hair (Benign) | Distichiasis | Verified |  |  | 1 |  |  |  |
|  | Hand (Benign) | Clinodactyly | Verified |  |  | 1 |  |  |  |
|  | Nails (Benign) | Hypoplastic nails | Verified |  |  | 1 |  |  |  |
|  | Nose (Benign) | Choanal atresia | Verified |  |  | 1 |  |  |  |
|  | Orofacial Cleft | Cleft palate | Verified |  |  | 1 | 1 | 1 | 1 |
|  | Ovarian Cancer |  | No Association | 0 |  |  | 1 |  |  |
|  | Prostate Cancer |  | Uncertain |  |  | 1 |  |  | 1 |
|  | Teeth (Benign) |  | Verified |  |  | 1 |  |  |  |
|  | Thyroid (Benign) | Thyroid hypoplasia | Uncertain |  |  | 1 |  |  |  |
| *CHEK2* | Adrenal Cortical Carcinoma |  | No Association |  |  |  |  | 1 | 1 |
|  | Breast Cancer |  | Verified | 1 | 1 | 1 | 1 | 1 | 1 |
|  | Colorectal Cancer |  | Verified | 9 | 1 | 1 | 1 |  |  |
|  | Gastric Cancer |  | Verified |  |  |  |  |  |  |
|  | Kidney Cancer |  | Verified |  |  |  |  |  |  |
|  | Leukemia | CLL | Uncertain |  |  |  |  | 1 | 1 |
|  | Lung Cancer |  | No Association |  |  |  | 1 |  |  |
|  | Ovarian Cancer |  | Uncertain |  |  |  | 1 |  |  |
|  | Prostate Cancer |  | Verified |  | 1 | 1 | 1 | 1 | 1 |
|  | Sarcoma (Bone) | Osteosarcoma | Verified |  |  |  | 1 | 1 | 1 |
|  | Thyroid Cancer | Papillary Thyroid Cancer | Verified |  |  |  |  |  |  |
| *NF1* | Abnormal Facies |  | Uncertain |  |  | 1 |  |  |  |
|  | Blood Vessel (Benign) | Renal artery stenosis | Uncertain |  |  | 1 |  |  |  |
|  | Bone (Benign) | Bone Dysplasia | Verified | 1 |  | 1 |  |  |  |
|  | Brain Tumor | Astrocytoma | Uncertain |  |  | 1 |  |  |  |
|  | Brain Tumor | Hypothalamic tumor | Uncertain |  |  | 1 |  |  |  |
|  | Brain Tumor | Meningioma | Uncertain |  |  | 1 |  |  |  |
|  | Brain Tumor | Optic Glioma | Verified | 1 | 1 | 1 |  |  |  |
|  | Breast Cancer |  | Verified |  | 1 |  |  |  |  |
|  | Cafe-Au-Lait Spots |  | Verified | 1 |  | 1 |  | 1 | 1 |
|  | Congenital Heart Disease | Pulmonary Stenosis | Verified |  |  | 1 |  | 1 | 1 |
|  | Eye (Benign) | Iris Hamartoma | Verified | 1 |  | 1 |  |  |  |
|  | GIST |  | Verified |  | 1 |  |  |  |  |
|  | Intellectual Disability |  | Verified | 1 |  | 1 |  |  |  |
|  | Leukemia |  | Verified |  |  | 1 | 1 | 1 | 1 |
|  | Neuroendocrine | Duodenal carcinoid | Uncertain |  |  | 1 |  |  |  |
|  | Neurofibroma |  | Verified | 1 | 1 | 1 | 1 | 1 | 1 |
|  | Ovarian Cancer |  | No Association | 0 |  |  |  |  |  |
|  | Paraganglioma |  | Verified | 1 |  |  |  |  |  |
|  | Parathyroid Neoplasm | Parathyroid Adenoma | Uncertain |  |  | 1 |  |  |  |
|  | Pheochromocytoma |  | Verified | 1 |  | 1 |  |  |  |
|  | Sarcoma | Malignant Peripheral  Nerve Sheath Tumor | Verified | 1 | 1 | 1 |  |  |  |
|  | Skin (Benign) | Skin Freckling | Verified | 1 |  | 1 |  |  |  |
| *PTEN* | Abnormal Facies |  | Uncertain |  |  | 1 |  |  |  |
|  | Autism |  | Verified |  | 1 | 1 | 1 | 1 | 1 |
|  | Bladder Cancer |  | Uncertain |  |  | 1 |  |  |  |
|  | Brain Tumor |  | Verified | 1 |  |  |  |  | 1 |
|  | Brain Tumor | Cerebellar Gangliocytoma | Verified |  | 1 | 1 |  |  |  |
|  | Brain Tumor | Meningioma | Uncertain |  |  | 1 |  |  |  |
|  | Breast Cancer |  | Verified | 1 | 1 | 1 | 1 |  |  |
|  | Breast (Benign) |  | Uncertain |  |  | 1 |  |  |  |
|  | Central Nervous System (Benign) | Cerebrovascular Malformations | Verified | 1 | 1 | 1 | 1 |  |  |
|  | Cervical Cancer |  | Uncertain |  |  | 1 |  |  |  |
|  | Colorectal Cancer |  | Verified | 1 | 1 |  |  |  |  |
|  | Ear (Benign) | Hearing Loss | Uncertain |  |  | 1 |  |  |  |
|  | Endometrial Cancer |  | Verified | 1 | 1 | 1 |  |  |  |
|  | Eye (Benign) | Cataract | Uncertain |  |  | 1 |  |  |  |
|  | Facial Papules |  | Verified | 1 | 1 | 1 |  |  |  |
|  | Gastric Cancer |  | Uncertain | 9 | 9 |  |  |  |  |
|  | Genitourinary (Benign) | Hydrocele | Uncertain |  |  | 1 |  |  |  |
|  | GI Neoplasm | Hamartomatous Polyps | Verified | 1 | 1 | 1 |  |  |  |
|  | Head and Neck (Benign) | Oral Mucosal Papillomatosis | Verified |  | 1 | 1 |  |  |  |
|  | Head and Neck Cancer | Squamous Cell Carcinoma | Uncertain |  |  |  | 1 |  |  |
|  | Kidney Cancer | Renal Cell Carcinoma | Verified | 1 | 1 |  |  |  |  |
|  | Lipoma |  | Verified | 1 | 1 | 1 |  |  |  |
|  | Liver (Benign) | Hepatomegaly | Uncertain |  |  | 1 |  |  |  |
|  | Lung Cancer |  | Uncertain |  |  |  | 1 |  |  |
|  | Macrocephaly |  | Verified | 1 | 1 | 1 |  | 1 | 1 |
|  | Melanoma |  | Verified | 1 |  |  |  |  |  |
|  | Oropharynx (Benign) |  | Uncertain |  |  | 1 |  |  |  |
|  | Ovarian Cancer |  | No Association |  |  |  | 1 |  |  |
|  | Prostate Cancer |  | Uncertain |  |  | 1 |  |  | 1 |
|  | Skin (Benign) | Acral Keratoses | Verified | 1 | 1 | 1 |  |  |  |
|  | Skin (Benign) | Macular Pigmentation  of Glans Penis | Verified | 1 | 1 |  |  |  |  |
|  | Skin (Benign) | Palmoplantar Keratoses | Verified |  | 1 | 1 |  |  |  |
|  | Skin (Benign) | Trichilemmoma | Verified | 1 | 1 | 1 |  |  |  |
|  | Skin Cancer (Non-Melanoma) | Squamous Cell Carcinoma | Uncertain |  |  |  |  | 1 |  |
|  | Spleen (Benign) | Splenomegaly | Uncertain |  |  | 1 |  |  |  |
|  | Thyroid (Benign) |  | Verified |  | 1 | 1 |  |  |  |
|  | Thyroid Cancer |  | Verified | 1 | 1 | 1 |  |  |  |
|  | Uterine Corpus (Benign) | Uterine Fibroids | Verified |  | 1 | 1 |  |  |  |
| *PALB2* | Breast Cancer |  | Verified | 1 | 1 | 1 | 1 | 1 | 1 |
|  | Colorectal Cancer |  | Uncertain | 9 | 1 |  |  |  |  |
|  | Gastrointestinal (Benign) | Tracheoesophageal Fistula | Uncertain |  |  |  |  | 1 |  |
|  | Ovarian Cancer |  | Verified | 1 | 1 |  | 1 |  |  |
|  | Pancreatic Cancer |  | Verified |  | 1 | 1 | 1 | 1 | 1 |
|  | Prostate Cancer |  | Verified |  | 1 |  |  |  |  |
| *RECQL* | Breast Cancer |  | Verified | 1 |  |  |  |  |  |
|  | Ovarian Cancer |  | No Association | 0 |  |  |  |  |  |
| *STK11* | Bladder (Benign) | Bladder Polyps | Uncertain |  |  | 1 |  |  |  |
|  | Breast Cancer |  | Verified | 1 | 1 | 1 | 1 |  |  |
|  | Cervical Cancer | Cervical Adenoma Malignum | Verified | 1 | 1 |  |  |  |  |
|  | Colorectal Cancer |  | Verified | 1 | 1 |  |  |  |  |
|  | Endometrial Cancer |  | Verified | 1 | 1 | 1 |  |  |  |
|  | Gastric Cancer |  | Verified | 1 | 1 | 1 |  |  |  |
|  | GI Hamartomatous Polyps |  | Verified | 1 | 1 |  | 1 | 1 |  |
|  | Hepatobiliary Cancer |  | Verified |  | 1 |  |  |  |  |
|  | Lung Cancer |  | Verified | 1 | 1 | 1 | 1 | 1 |  |
|  | Nose (Benign) | Nasal Polyps | Uncertain |  |  | 1 |  |  |  |
|  | Ovarian Cancer |  | No Association | 0 |  |  | 1 |  |  |
|  | Ovarian Neoplasm | Non-Epithelial ovarian tumor | Verified |  | 1 |  |  |  |  |
|  | Ovarian Neoplasm | Sex Cord Tumor | Verified |  | 1 |  |  |  |  |
|  | Pancreatic Cancer |  | Verified | 1 | 1 |  |  | 1 | 1 |
|  | Respiratory (Benign) | Bronchial Polyps | Uncertain |  |  | 1 |  |  |  |
|  | Skin (Benign) | Perioral Freckling | Verified | 1 | 1 | 1 |  | 1 |  |
|  | Small Intestine Cancer |  | Verified | 1 | 1 |  |  |  |  |
|  | Testicular Neoplasm | Sertoli Cell Tumor | Verified | 1 | 1 |  |  | 1 | 1 |
|  | Thyroid Cancer |  | No Association |  |  | 1 |  |  |  |
| *TP53* | Adrenal Cortical Carcinoma |  | Verified |  | 1 | 1 |  | 1 | 1 |
|  | Bone (Benign) | Short Stature | Uncertain |  |  | 1 |  |  |  |
|  | Brain Tumor | Choroid Plexus Papilloma | Verified |  | 1 | 1 |  | 1 |  |
|  | Brain Tumor | Glioma | Verified |  |  | 1 | 1 |  | 1 |
|  | Brain Tumor | Medulloblastoma | Verified |  | 1 |  |  |  |  |
|  | Breast Cancer |  | Verified |  | 1 |  | 1 | 1 | 1 |
|  | Colorectal Cancer |  | Verified | 1 | 1 | 1 |  |  | 1 |
|  | Gastric Cancer |  | Uncertain |  | 9 | 1 |  |  |  |
|  | Head and Neck Cancer |  | No Association |  |  |  | 1 |  |  |
|  | Hepatobiliary Cancer | Hepatocellular Cancer | Verified | 1 |  |  |  |  | 1 |
|  | Lung Cancer |  | Uncertain |  |  | 1 |  |  |  |
|  | Melanoma |  | Uncertain |  |  |  | 1 |  |  |
|  | Nasopharyngeal Cancer |  | Uncertain |  |  |  |  |  | 1 |
|  | Ovarian Cancer |  | Uncertain | 9 |  |  |  |  |  |
|  | Pancreatic Cancer |  | Verified | 1 |  | 1 |  |  | 1 |
|  | Prostate Cancer |  | No Association |  |  | 1 |  |  |  |
|  | Sarcoma | Osteosarcoma | Verified |  | 1 | 1 | 1 | 1 | 1 |
|  | Sarcoma | Soft Tissue Sarcoma | Verified |  | 1 | 1 | 1 | 1 |  |
|  | Skin Cancer (Non-Melanoma) | Basal Cell Carcinoma | Uncertain |  |  | 1 |  |  | 1 |
|  | Wilms Tumor |  | Uncertain |  |  |  | 1 |  |  |

Abbreviations: GHR, GeneticsHomeRefence; NSCLC, non-small cell lung cancer; CLL, chronic lymphocytic leukemia

Note: The number ‘1’ indicates that the gene was associated with the disease/cancer in the resource. The number ‘0’ indicates that the gene’s association with the disease/cancer was refuted in the resource. The number ‘9’ indicates that the gene’s association with the disease/cancer was unclear in the resource. Blank space indicates association was not found in the resource.
