# Supplementary material for: Disease Spectrum of Breast Cancer Susceptibility Genes
Source: Front Oncol. 2021 Apr 20;11:663419. doi: 10.3389/fonc.2021.663419 (PMC8093501; doi:10.3389/fonc.2021.663419)
Supplement: Supplementary file 2 [file Table_2.docx]

**Supplementary Table 2. Search terms of gene-disease association for NLP**

| **Genes** | **Diseases** | **Predominant Subtype** | **SearchTerms** |
| --- | --- | --- | --- |
| *ATM* | Brain Tumor | Glioblastoma Multiforme | (Ataxia Telangiectasia Mutated Proteins [MESH] OR Ataxia Telangiectasia [MESH] OR AT mutated [TIAB] OR AT protein [TIAB] OR AT1 [TIAB] OR ATA [TIAB] OR ataxia telangiectasia mutated [TIAB] OR ataxia telangiectasia mutated protein [TIAB] OR ataxia telangiectasia mutated (includes complementation groups A, C and D) [TIAB] OR ATD [TIAB] OR ATC [TIAB] OR ATDC [TIAB] OR ATE [TIAB] OR ATM_HUMAN [TIAB] OR human phosphatidylinositol 3-kinase homolog [TIAB] OR serine-protein kinase ATM [TIAB] OR TEL1 [TIAB] OR TELO1 [TIAB] OR EC 2.7.11.1 [TIAB] OR ATM Serine/Threonine Kinase [TIAB] OR ataxia-telangiectasia (A-T) gene [TIAB] OR ATM [TIAB] OR hATM [TIAB] OR A-T mutation [TIAB]) AND ("Brain Neoplasms"[Mesh] OR ((Brain[tiab] OR central nervous system[tiab] OR cerebral hemispheres[tiab] OR basal ganglia[tiab] OR hypothalamus[tiab] OR thalamus[tiab] OR brain stem[tiab] OR cerebellum[taib]) AND (cancer*[tiab] OR carcinoma*[tiab] OR neoplasm*[tiab] OR neoplasia*[tiab] OR tumor*[tiab] OR tumour*[tiab])) OR "Glioblastoma Multiforme") |
|  | Gastric Cancer |  | (Ataxia Telangiectasia Mutated Proteins [MESH] OR Ataxia Telangiectasia [MESH] OR AT mutated [TIAB] OR AT protein [TIAB] OR AT1 [TIAB] OR ATA [TIAB] OR ataxia telangiectasia mutated [TIAB] OR ataxia telangiectasia mutated protein [TIAB] OR ataxia telangiectasia mutated (includes complementation groups A, C and D) [TIAB] OR ATD [TIAB] OR ATC [TIAB] OR ATDC [TIAB] OR ATE [TIAB] OR ATM_HUMAN [TIAB] OR human phosphatidylinositol 3-kinase homolog [TIAB] OR serine-protein kinase ATM [TIAB] OR TEL1 [TIAB] OR TELO1 [TIAB] OR EC 2.7.11.1 [TIAB] OR ATM Serine/Threonine Kinase [TIAB] OR ataxia-telangiectasia (A-T) gene [TIAB] OR ATM [TIAB] OR hATM [TIAB] OR A-T mutation [TIAB]) AND ((Stomach[tiab] OR Gastric[tiab]) AND (cancer*[tiab] OR carcinoma*[tiab] OR neoplasm*[tiab] OR neoplasia*[tiab] OR tumor*[tiab] OR tumour*[tiab]))) |
|  | Lung Cancer | NSCLC | (Ataxia Telangiectasia Mutated Proteins [MESH] OR Ataxia Telangiectasia [MESH] OR AT mutated [TIAB] OR AT protein [TIAB] OR AT1 [TIAB] OR ATA [TIAB] OR ataxia telangiectasia mutated [TIAB] OR ataxia telangiectasia mutated protein [TIAB] OR ataxia telangiectasia mutated (includes complementation groups A, C and D) [TIAB] OR ATD [TIAB] OR ATC [TIAB] OR ATDC [TIAB] OR ATE [TIAB] OR ATM_HUMAN [TIAB] OR human phosphatidylinositol 3-kinase homolog [TIAB] OR serine-protein kinase ATM [TIAB] OR TEL1 [TIAB] OR TELO1 [TIAB] OR EC 2.7.11.1 [TIAB] OR ATM Serine/Threonine Kinase [TIAB] OR ataxia-telangiectasia (A-T) gene [TIAB] OR ATM [TIAB] OR hATM [TIAB] OR A-T mutation [TIAB]) AND ("Lung Neoplasms"[Mesh] OR ((Lung[tiab] OR Pulmonary[tiab]) AND (cancer*[tiab] OR carcinoma*[tiab] OR neoplasm*[tiab] OR neoplasia*[tiab] OR tumor*[tiab] OR tumour*[tiab]))) |
|  | Melanoma |  | (Ataxia Telangiectasia Mutated Proteins [MESH] OR Ataxia Telangiectasia [MESH] OR AT mutated [TIAB] OR AT protein [TIAB] OR AT1 [TIAB] OR ATA [TIAB] OR ataxia telangiectasia mutated [TIAB] OR ataxia telangiectasia mutated protein [TIAB] OR ataxia telangiectasia mutated (includes complementation groups A, C and D) [TIAB] OR ATD [TIAB] OR ATC [TIAB] OR ATDC [TIAB] OR ATE [TIAB] OR ATM_HUMAN [TIAB] OR human phosphatidylinositol 3-kinase homolog [TIAB] OR serine-protein kinase ATM [TIAB] OR TEL1 [TIAB] OR TELO1 [TIAB] OR EC 2.7.11.1 [TIAB] OR ATM Serine/Threonine Kinase [TIAB] OR ataxia-telangiectasia (A-T) gene [TIAB] OR ATM [TIAB] OR hATM [TIAB] OR A-T mutation [TIAB]) AND ("Melanoma" [TIAB] OR " Melanoma" [Mesh]) |
|  | Ovarian Cancer |  | (Ataxia Telangiectasia Mutated Proteins [MESH] OR Ataxia Telangiectasia [MESH] OR AT mutated [TIAB] OR AT protein [TIAB] OR AT1 [TIAB] OR ATA [TIAB] OR ataxia telangiectasia mutated [TIAB] OR ataxia telangiectasia mutated protein [TIAB] OR ataxia telangiectasia mutated (includes complementation groups A, C and D) [TIAB] OR ATD [TIAB] OR ATC [TIAB] OR ATDC [TIAB] OR ATE [TIAB] OR ATM_HUMAN [TIAB] OR human phosphatidylinositol 3-kinase homolog [TIAB] OR serine-protein kinase ATM [TIAB] OR TEL1 [TIAB] OR TELO1 [TIAB] OR EC 2.7.11.1 [TIAB] OR ATM Serine/Threonine Kinase [TIAB] OR ataxia-telangiectasia (A-T) gene [TIAB] OR ATM [TIAB] OR hATM [TIAB] OR A-T mutation [TIAB]) AND ("Ovarian Neoplasms"[Mesh] OR ((ovary[tiab] OR ovaries[tiab] OR ovarian[taib]) AND (cancer*[tiab] OR carcinoma*[tiab] OR neoplasm*[tiab] OR neoplasia*[tiab] OR tumor*[tiab] OR tumour*[tiab]))) |
| *BARD1* | Colorectal Cancer |  | (BARD1 protein, human [Supplementary Concept] OR BRCA1 Associated RING Domain 1 [TIAB] OR RING-Type E3 Ubiquitin Transferase BARD1 [TIAB] OR BARD-1 [TIAB] OR EC 2.3.2.27 [TIAB] OR BARD1 [TIAB] OR hBARD1 [TIAB]) AND ("Colorectal Neoplasms"[Mesh] OR (Colorectal[tiab] AND (cancer*[tiab] OR carcinoma*[tiab] OR neoplasm*[tiab] OR neoplasia*[tiab] OR tumor*[tiab] OR tumour*[tiab]))) |
|  | Ovarian Cancer |  | (BARD1 protein, human [Supplementary Concept] OR BRCA1 Associated RING Domain 1 [TIAB] OR RING-Type E3 Ubiquitin Transferase BARD1 [TIAB] OR BARD-1 [TIAB] OR EC 2.3.2.27 [TIAB] OR BARD1 [TIAB] OR hBARD1 [TIAB]) AND ("Ovarian Neoplasms"[Mesh] OR ((ovary[tiab] OR ovaries[tiab] OR ovarian[taib]) AND (cancer*[tiab] OR carcinoma*[tiab] OR neoplasm*[tiab] OR neoplasia*[tiab] OR tumor*[tiab] OR tumour*[tiab]))) |
| *BRCA1* | Colorectal Cancer |  | (Genes, BRCA1 [MESH] OR BRCC1 [TIAB] OR breast cancer 1 gene [TIAB] OR breast cancer 1, early onset gene [TIAB] OR breast cancer type 1 susceptibility gene [TIAB] OR breast cancer type 1 susceptibility protein [TIAB] OR IRIS [TIAB] OR PPP1R53 [TIAB] OR PSCP [TIAB] OR RNF53 [TIAB] OR BRCA1, DNA Repair Associated [TIAB] OR Protein Phosphatase 1, Regulatory Subunit 53 [TIAB] OR BRCA1/BRCA2-Containing Complex, Subunit 1 [TIAB] OR Fanconi Anemia, Complementation Group S [TIAB] OR RING Finger Protein 53 [TIAB] OR BRCA1_HUMAN [TIAB] OR Breast And Ovarian Cancer Susceptibility Protein 1 [TIAB] OR RING-Type E3 Ubiquitin Transferase BRCA1 [TIAB] OR EC 2.3.2.27 [TIAB] OR EC 6.3.2 [TIAB] OR BROVCA1 [TIAB] OR BRCAI [TIAB] OR FANCS [TIAB] OR PNCA4 [TIAB] OR BRCA1 [TIAB] OR 17q-linked [TIAB] OR hBRCA1 [TIAB] OR BRCA1 gene [TIAB] OR breast cancer 1 [TIAB] OR breast cancer 1, early onset [TIAB]) AND ("Colorectal Neoplasms"[Mesh] OR (Colorectal[tiab] AND (cancer*[tiab] OR carcinoma*[tiab] OR neoplasm*[tiab] OR neoplasia*[tiab] OR tumor*[tiab] OR tumour*[tiab]))) |
|  | Gastric Cancer |  | (Genes, BRCA1 [MESH] OR BRCC1 [TIAB] OR breast cancer 1 gene [TIAB] OR breast cancer 1, early onset gene [TIAB] OR breast cancer type 1 susceptibility gene [TIAB] OR breast cancer type 1 susceptibility protein [TIAB] OR IRIS [TIAB] OR PPP1R53 [TIAB] OR PSCP [TIAB] OR RNF53 [TIAB] OR BRCA1, DNA Repair Associated [TIAB] OR Protein Phosphatase 1, Regulatory Subunit 53 [TIAB] OR BRCA1/BRCA2-Containing Complex, Subunit 1 [TIAB] OR Fanconi Anemia, Complementation Group S [TIAB] OR RING Finger Protein 53 [TIAB] OR BRCA1_HUMAN [TIAB] OR Breast And Ovarian Cancer Susceptibility Protein 1 [TIAB] OR RING-Type E3 Ubiquitin Transferase BRCA1 [TIAB] OR EC 2.3.2.27 [TIAB] OR EC 6.3.2 [TIAB] OR BROVCA1 [TIAB] OR BRCAI [TIAB] OR FANCS [TIAB] OR PNCA4 [TIAB] OR BRCA1 [TIAB] OR 17q-linked [TIAB] OR hBRCA1 [TIAB] OR BRCA1 gene [TIAB] OR breast cancer 1 [TIAB] OR breast cancer 1, early onset [TIAB]) AND ((Stomach[tiab] OR Gastric[tiab]) AND (cancer*[tiab] OR carcinoma*[tiab] OR neoplasm*[tiab] OR neoplasia*[tiab] OR tumor*[tiab] OR tumour*[tiab]))) |
| *CDH1* | Central Nervous System (Benign) | Neural tube defect | (Cdh1 Proteins/genetics [MESH] OR Arc-1 [TIAB] OR CADH1_HUMAN [TIAB] OR cadherin 1, type 1 [TIAB] OR Calcium-Dependent Adhesion Protein, Epithelial [TIAB] OR CAM 120/80 [TIAB] OR CD324 [TIAB] OR CDHE [TIAB] OR Cell-CAM 120/80 [TIAB] OR ECAD [TIAB] OR LCAM [TIAB] OR liver cell adhesion molecule [TIAB] OR UVO [TIAB] OR uvomorulin [TIAB] OR cadherin 1, type 1, E-cadherin (epithelial) [TIAB] OR CD324 Antigen [TIAB] OR epithelial cadherin [TIAB] OR CDH1 [TIAB] OR e-cadherin [TIAB] OR epithelial-cadherin [TIAB] OR cadherin [TIAB] OR hCDH1 [TIAB] OR cadherin 1, E-cadherin [TIAB] OR cadherin 1, type 1, E-cadherin [TIAB]) AND ("Neural Tube Defects"[Mesh] OR ((Neural Tube[tiab] AND Defect[tiab]) OR (Craniorachischises[tiab]) OR (Diastematomyelias[tiab]) OR (Spinal Dysraphism[tiab]) OR (Myelodysplasia[tiab] )) |
|  | Colorectal Cancer |  | (Cdh1 Proteins/genetics [MESH] OR Arc-1 [TIAB] OR CADH1_HUMAN [TIAB] OR cadherin 1, type 1 [TIAB] OR Calcium-Dependent Adhesion Protein, Epithelial [TIAB] OR CAM 120/80 [TIAB] OR CD324 [TIAB] OR CDHE [TIAB] OR Cell-CAM 120/80 [TIAB] OR ECAD [TIAB] OR LCAM [TIAB] OR liver cell adhesion molecule [TIAB] OR UVO [TIAB] OR uvomorulin [TIAB] OR cadherin 1, type 1, E-cadherin (epithelial) [TIAB] OR CD324 Antigen [TIAB] OR epithelial cadherin [TIAB] OR CDH1 [TIAB] OR e-cadherin [TIAB] OR epithelial-cadherin [TIAB] OR cadherin [TIAB] OR hCDH1 [TIAB] OR cadherin 1, E-cadherin [TIAB] OR cadherin 1, type 1, E-cadherin [TIAB]) AND ("Colorectal Neoplasms"[Mesh] OR (Colorectal[tiab] AND (cancer*[tiab] OR carcinoma*[tiab] OR neoplasm*[tiab] OR neoplasia*[tiab] OR tumor*[tiab] OR tumour*[tiab]))) |
|  | Endometrial Cancer |  | (Cdh1 Proteins/genetics [MESH] OR Arc-1 [TIAB] OR CADH1_HUMAN [TIAB] OR cadherin 1, type 1 [TIAB] OR Calcium-Dependent Adhesion Protein, Epithelial [TIAB] OR CAM 120/80 [TIAB] OR CD324 [TIAB] OR CDHE [TIAB] OR Cell-CAM 120/80 [TIAB] OR ECAD [TIAB] OR LCAM [TIAB] OR liver cell adhesion molecule [TIAB] OR UVO [TIAB] OR uvomorulin [TIAB] OR cadherin 1, type 1, E-cadherin (epithelial) [TIAB] OR CD324 Antigen [TIAB] OR epithelial cadherin [TIAB] OR CDH1 [TIAB] OR e-cadherin [TIAB] OR epithelial-cadherin [TIAB] OR cadherin [TIAB] OR hCDH1 [TIAB] OR cadherin 1, E-cadherin [TIAB] OR cadherin 1, type 1, E-cadherin [TIAB]) AND ("Endometrial Neoplasms"[Mesh] OR ((Endometrial [tiab] OR Endometrium[tiab] ) AND (cancer*[tiab] OR carcinoma*[tiab] OR neoplasm*[tiab] OR neoplasia*[tiab] OR tumor*[tiab] OR tumour*[tiab]))) |
|  | Ovarian Cancer |  | (Cdh1 Proteins/genetics [MESH] OR Arc-1 [TIAB] OR CADH1_HUMAN [TIAB] OR cadherin 1, type 1 [TIAB] OR Calcium-Dependent Adhesion Protein, Epithelial [TIAB] OR CAM 120/80 [TIAB] OR CD324 [TIAB] OR CDHE [TIAB] OR Cell-CAM 120/80 [TIAB] OR ECAD [TIAB] OR LCAM [TIAB] OR liver cell adhesion molecule [TIAB] OR UVO [TIAB] OR uvomorulin [TIAB] OR cadherin 1, type 1, E-cadherin (epithelial) [TIAB] OR CD324 Antigen [TIAB] OR epithelial cadherin [TIAB] OR CDH1 [TIAB] OR e-cadherin [TIAB] OR epithelial-cadherin [TIAB] OR cadherin [TIAB] OR hCDH1 [TIAB] OR cadherin 1, E-cadherin [TIAB] OR cadherin 1, type 1, E-cadherin [TIAB]) AND ("Ovarian Neoplasms"[Mesh] OR ((ovary[tiab] OR ovaries[tiab] OR ovarian[taib]) AND (cancer*[tiab] OR carcinoma*[tiab] OR neoplasm*[tiab] OR neoplasia*[tiab] OR tumor*[tiab] OR tumour*[tiab]))) |
|  | Thyroid (Benign) | Thyroid hypoplasia | (Cdh1 Proteins/genetics [MESH] OR Arc-1 [TIAB] OR CADH1_HUMAN [TIAB] OR cadherin 1, type 1 [TIAB] OR Calcium-Dependent Adhesion Protein, Epithelial [TIAB] OR CAM 120/80 [TIAB] OR CD324 [TIAB] OR CDHE [TIAB] OR Cell-CAM 120/80 [TIAB] OR ECAD [TIAB] OR LCAM [TIAB] OR liver cell adhesion molecule [TIAB] OR UVO [TIAB] OR uvomorulin [TIAB] OR cadherin 1, type 1, E-cadherin (epithelial) [TIAB] OR CD324 Antigen [TIAB] OR epithelial cadherin [TIAB] OR CDH1 [TIAB] OR e-cadherin [TIAB] OR epithelial-cadherin [TIAB] OR cadherin [TIAB] OR hCDH1 [TIAB] OR cadherin 1, E-cadherin [TIAB] OR cadherin 1, type 1, E-cadherin [TIAB]) AND ("Thyroid Dysgenesis"[Mesh] OR (Thyroid[tiab] AND (Dysgenesis[tiab]) OR Hypoplasia[tiab] OR Ectopic[tiab] ORAgenesis[tiab] ))) |
| *CHEK2* | Adrenal Cortical Carcinoma |  | (CHEK2 protein, human [Supplementary Concept] OR CDS1 [TIAB] OR Cds1 kinase [TIAB] OR Checkpoint-Like Protein CHK2 [TIAB] OR CHK2 checkpoint homolog (S. pombe) [TIAB] OR Chk2 protein kinase [TIAB] OR CHK2_HUMAN [TIAB] OR hCds1 protein [TIAB] OR hCHK2 [TIAB] OR HuCds1 [TIAB] OR RAD53 [TIAB] OR Serine/Threonine-Protein Kinase CHK2 [TIAB] OR HCds1 [TIAB] OR CHK2 (Checkpoint, S.Pombe) Homolog [TIAB] OR EC 2.7.11.1 [TIAB] OR EC 2.7.11 [TIAB] OR PP1425 [TIAB] OR LFS2 [TIAB] OR CHEK2 [TIAB] OR Checkpoint kinase 2 [TIAB] OR CHK2 [TIAB] OR hCHEK2 [TIAB] OR cell cycle checkpoint kinase 2 [TIAB] OR CHK2 checkpoint homolog [TIAB]) AND ("Adrenocortical Carcinoma"[Mesh] OR ((Adrenocortical[tiab] OR Adrenal Cortical[tiab]) AND (Carcinoma[tiab] OR neoplasm*[tiab] OR neoplasia*[tiab] OR tumor*[tiab] OR tumour*[tiab]))) |
|  | Gastric Cancer |  | (CHEK2 protein, human [Supplementary Concept] OR CDS1 [TIAB] OR Cds1 kinase [TIAB] OR Checkpoint-Like Protein CHK2 [TIAB] OR CHK2 checkpoint homolog (S. pombe) [TIAB] OR Chk2 protein kinase [TIAB] OR CHK2_HUMAN [TIAB] OR hCds1 protein [TIAB] OR hCHK2 [TIAB] OR HuCds1 [TIAB] OR RAD53 [TIAB] OR Serine/Threonine-Protein Kinase CHK2 [TIAB] OR HCds1 [TIAB] OR CHK2 (Checkpoint, S.Pombe) Homolog [TIAB] OR EC 2.7.11.1 [TIAB] OR EC 2.7.11 [TIAB] OR PP1425 [TIAB] OR LFS2 [TIAB] OR CHEK2 [TIAB] OR Checkpoint kinase 2 [TIAB] OR CHK2 [TIAB] OR hCHEK2 [TIAB] OR cell cycle checkpoint kinase 2 [TIAB] OR CHK2 checkpoint homolog [TIAB]) AND ((Stomach[tiab] OR Gastric[tiab]) AND (cancer*[tiab] OR carcinoma*[tiab] OR neoplasm*[tiab] OR neoplasia*[tiab] OR tumor*[tiab] OR tumour*[tiab]))) |
|  | Kidney Cancer |  | (CHEK2 protein, human [Supplementary Concept] OR CDS1 [TIAB] OR Cds1 kinase [TIAB] OR Checkpoint-Like Protein CHK2 [TIAB] OR CHK2 checkpoint homolog (S. pombe) [TIAB] OR Chk2 protein kinase [TIAB] OR CHK2_HUMAN [TIAB] OR hCds1 protein [TIAB] OR hCHK2 [TIAB] OR HuCds1 [TIAB] OR RAD53 [TIAB] OR Serine/Threonine-Protein Kinase CHK2 [TIAB] OR HCds1 [TIAB] OR CHK2 (Checkpoint, S.Pombe) Homolog [TIAB] OR EC 2.7.11.1 [TIAB] OR EC 2.7.11 [TIAB] OR PP1425 [TIAB] OR LFS2 [TIAB] OR CHEK2 [TIAB] OR Checkpoint kinase 2 [TIAB] OR CHK2 [TIAB] OR hCHEK2 [TIAB] OR cell cycle checkpoint kinase 2 [TIAB] OR CHK2 checkpoint homolog [TIAB]) AND ("Kidney Neoplasms"[Mesh] OR ((kidney [TIAB] OR renal [TIAB]) AND (cancer*[TIAB] OR carcinoma*[TIAB] OR neoplasm*[TIAB] OR neoplasia*[TIAB] OR tumor*[TIAB] OR tumour*[TIAB]))) |
|  | Leukemia | CLL | (CHEK2 protein, human [Supplementary Concept] OR CDS1 [TIAB] OR Cds1 kinase [TIAB] OR Checkpoint-Like Protein CHK2 [TIAB] OR CHK2 checkpoint homolog (S. pombe) [TIAB] OR Chk2 protein kinase [TIAB] OR CHK2_HUMAN [TIAB] OR hCds1 protein [TIAB] OR hCHK2 [TIAB] OR HuCds1 [TIAB] OR RAD53 [TIAB] OR Serine/Threonine-Protein Kinase CHK2 [TIAB] OR HCds1 [TIAB] OR CHK2 (Checkpoint, S.Pombe) Homolog [TIAB] OR EC 2.7.11.1 [TIAB] OR EC 2.7.11 [TIAB] OR PP1425 [TIAB] OR LFS2 [TIAB] OR CHEK2 [TIAB] OR Checkpoint kinase 2 [TIAB] OR CHK2 [TIAB] OR hCHEK2 [TIAB] OR cell cycle checkpoint kinase 2 [TIAB] OR CHK2 checkpoint homolog [TIAB]) AND ("Leukemia" [TIAB] OR "Leukemia" [Mesh]) |
|  | Lung Cancer |  | (CHEK2 protein, human [Supplementary Concept] OR CDS1 [TIAB] OR Cds1 kinase [TIAB] OR Checkpoint-Like Protein CHK2 [TIAB] OR CHK2 checkpoint homolog (S. pombe) [TIAB] OR Chk2 protein kinase [TIAB] OR CHK2_HUMAN [TIAB] OR hCds1 protein [TIAB] OR hCHK2 [TIAB] OR HuCds1 [TIAB] OR RAD53 [TIAB] OR Serine/Threonine-Protein Kinase CHK2 [TIAB] OR HCds1 [TIAB] OR CHK2 (Checkpoint, S.Pombe) Homolog [TIAB] OR EC 2.7.11.1 [TIAB] OR EC 2.7.11 [TIAB] OR PP1425 [TIAB] OR LFS2 [TIAB] OR CHEK2 [TIAB] OR Checkpoint kinase 2 [TIAB] OR CHK2 [TIAB] OR hCHEK2 [TIAB] OR cell cycle checkpoint kinase 2 [TIAB] OR CHK2 checkpoint homolog [TIAB]) AND ("Lung Neoplasms"[Mesh] OR ((Lung[tiab] OR Pulmonary[tiab]) AND (cancer*[tiab] OR carcinoma*[tiab] OR neoplasm*[tiab] OR neoplasia*[tiab] OR tumor*[tiab] OR tumour*[tiab]))) |
|  | Ovarian Cancer |  | (CHEK2 protein, human [Supplementary Concept] OR CDS1 [TIAB] OR Cds1 kinase [TIAB] OR Checkpoint-Like Protein CHK2 [TIAB] OR CHK2 checkpoint homolog (S. pombe) [TIAB] OR Chk2 protein kinase [TIAB] OR CHK2_HUMAN [TIAB] OR hCds1 protein [TIAB] OR hCHK2 [TIAB] OR HuCds1 [TIAB] OR RAD53 [TIAB] OR Serine/Threonine-Protein Kinase CHK2 [TIAB] OR HCds1 [TIAB] OR CHK2 (Checkpoint, S.Pombe) Homolog [TIAB] OR EC 2.7.11.1 [TIAB] OR EC 2.7.11 [TIAB] OR PP1425 [TIAB] OR LFS2 [TIAB] OR CHEK2 [TIAB] OR Checkpoint kinase 2 [TIAB] OR CHK2 [TIAB] OR hCHEK2 [TIAB] OR cell cycle checkpoint kinase 2 [TIAB] OR CHK2 checkpoint homolog [TIAB]) AND ("Ovarian Neoplasms"[Mesh] OR ((ovary[tiab] OR ovaries[tiab] OR ovarian[taib]) AND (cancer*[tiab] OR carcinoma*[tiab] OR neoplasm*[tiab] OR neoplasia*[tiab] OR tumor*[tiab] OR tumour*[tiab]))) |
|  | Thyroid Cancer |  | (CHEK2 protein, human [Supplementary Concept] OR CDS1 [TIAB] OR Cds1 kinase [TIAB] OR Checkpoint-Like Protein CHK2 [TIAB] OR CHK2 checkpoint homolog (S. pombe) [TIAB] OR Chk2 protein kinase [TIAB] OR CHK2_HUMAN [TIAB] OR hCds1 protein [TIAB] OR hCHK2 [TIAB] OR HuCds1 [TIAB] OR RAD53 [TIAB] OR Serine/Threonine-Protein Kinase CHK2 [TIAB] OR HCds1 [TIAB] OR CHK2 (Checkpoint, S.Pombe) Homolog [TIAB] OR EC 2.7.11.1 [TIAB] OR EC 2.7.11 [TIAB] OR PP1425 [TIAB] OR LFS2 [TIAB] OR CHEK2 [TIAB] OR Checkpoint kinase 2 [TIAB] OR CHK2 [TIAB] OR hCHEK2 [TIAB] OR cell cycle checkpoint kinase 2 [TIAB] OR CHK2 checkpoint homolog [TIAB]) AND ("Thyroid Neoplasms"[Mesh] OR (Thyroid[tiab] AND (cancer*[tiab] OR carcinoma*[tiab] OR neoplasm*[tiab] OR neoplasia*[tiab] OR tumor*[tiab] OR tumour*[tiab]))) |
| *NF1* | Blood Vessel (Benign) | Renal artery stenosis | (Neurofibromatosis 1 [MESH] OR NF1 [TIAB] OR Neurofibromatosis-related protein NF-1 [TIAB] OR Neurofibromatosis Type 1 Protein [TIAB] OR neurofibromin 1 [TIAB] OR NF1-GAP-Related Protein [TIAB] OR NF1 GRP [TIAB] OR NF1 Protein [TIAB] OR NF1_HUMAN [TIAB]) AND ("Renal Artery Obstruction"[Mesh] OR (Renal Artery[tiab] AND (Obstruction[tiab] OR Stenoses[tiab] OR Stenosis[tiab] ))) |
|  | Neuroendocrine | Duodenal carcinoid | (Neurofibromatosis 1 [MESH] OR NF1 [TIAB] OR Neurofibromatosis-related protein NF-1 [TIAB] OR Neurofibromatosis Type 1 Protein [TIAB] OR neurofibromin 1 [TIAB] OR NF1-GAP-Related Protein [TIAB] OR NF1 GRP [TIAB] OR NF1 Protein [TIAB] OR NF1_HUMAN [TIAB]) AND ("Neuroendocrine Neoplasms"[Mesh] OR (((Acidophil[tiab] OR Basophil[tiab] OR Chromophobe[tiab] OR basal ganglia[tiab]) AND Adenoma[tiab] ) OR Carcinoid Tumor[tiab] OR Neuroendocrine Carcinoma[tiab] OR (Paraganglioma[tiab] OR Paraganglioma[tiab] OR Pheochromocytoma[tiab]))) |
|  | Ovarian Cancer |  | (Neurofibromatosis 1 [MESH] OR NF1 [TIAB] OR Neurofibromatosis-related protein NF-1 [TIAB] OR Neurofibromatosis Type 1 Protein [TIAB] OR neurofibromin 1 [TIAB] OR NF1-GAP-Related Protein [TIAB] OR NF1 GRP [TIAB] OR NF1 Protein [TIAB] OR NF1_HUMAN [TIAB]) AND ("Ovarian Neoplasms"[Mesh] OR ((ovary[tiab] OR ovaries[tiab] OR ovarian[taib]) AND (cancer*[tiab] OR carcinoma*[tiab] OR neoplasm*[tiab] OR neoplasia*[tiab] OR tumor*[tiab] OR tumour*[tiab]))) |
|  | Parathyroid Neoplasm | Parathyroid Adenoma | (Neurofibromatosis 1 [MESH] OR NF1 [TIAB] OR Neurofibromatosis-related protein NF-1 [TIAB] OR Neurofibromatosis Type 1 Protein [TIAB] OR neurofibromin 1 [TIAB] OR NF1-GAP-Related Protein [TIAB] OR NF1 GRP [TIAB] OR NF1 Protein [TIAB] OR NF1_HUMAN [TIAB]) AND ("Parathyroid Neoplasms"[Mesh] OR (Parathyroid[tiab] AND (cancer*[tiab] OR carcinoma*[tiab] OR neoplasm*[tiab] OR neoplasia*[tiab] OR tumor*[tiab] OR tumour*[tiab]))) |
| *PALB2* | Colorectal Cancer |  | (PALB2 protein, human [Supplementary Concept] OR PNCA3 [TIAB] OR Fanconi Anemia, Complementation Group N [TIAB] OR FANCN [TIAB] OR Partner and Localizer Of BRCA2 [TIAB] OR PALB2 [TIAB] OR hPALB2 [TIAB]) AND ("Colorectal Neoplasms"[Mesh] OR (Colorectal[tiab] AND (cancer*[tiab] OR carcinoma*[tiab] OR neoplasm*[tiab] OR neoplasia*[tiab] OR tumor*[tiab] OR tumour*[tiab]))) |
|  | Gastrointestinal (Benign) | Tracheoesophageal Fistula | (PALB2 protein, human [Supplementary Concept] OR PNCA3 [TIAB] OR Fanconi Anemia, Complementation Group N [TIAB] OR FANCN [TIAB] OR Partner and Localizer Of BRCA2 [TIAB] OR PALB2 [TIAB] OR hPALB2 [TIAB]) AND ("Tracheoesophageal Fistula"[Mesh] OR ((Tracheoesophageal[tiab] OREsophagotracheal[tiab]) AND Fistula[tiab])) |
| *PTEN* | Bladder Cancer |  | (PTEN protein, human [Supplementary Concept] OR MMAC1 [TIAB] OR mutated in multiple advanced cancers 1 [TIAB] OR phosphatase and tensin homolog (mutated in multiple advanced cancers 1) [TIAB] OR phosphatase and tensin homolog deleted on chromosome 10 [TIAB] OR protein-tyrosine phosphatase PTEN [TIAB] OR PTEN-MMAC1 protein [TIAB] OR PTEN1 [TIAB] OR PTEN_HUMAN [TIAB] OR TEP1 [TIAB] OR TEP1 phosphatase [TIAB] OR Phosphatase And Tensin Homolog [TIAB] OR Phosphatidylinositol 3,4,5-Trisphosphate 3-Phosphatase And Dual-Specificity Protein Phosphatase PTEN [TIAB] OR Phosphatidylinositol-3,4,5-Trisphosphate 3-Phosphatase And Dual-Specificity Protein Phosphatase PTEN [TIAB] OR MMAC1 Phosphatase And Tensin Homolog Deleted On Chromosome 10 [TIAB] OR Mitochondrial Phosphatase And Tensin Protein Alpha [TIAB] OR Phosphatase And Tensin-Like Protein [TIAB] OR Mitochondrial PTENalpha [TIAB] OR EC 3.1.3.16 [TIAB] OR EC 3.1.3.48 [TIAB] OR EC 3.1.3.67 [TIAB] OR 10q23del [TIAB] OR PTENbeta [TIAB] OR GLM2 [TIAB] OR MHAM [TIAB] OR DEC [TIAB] OR BZS [TIAB] OR CWS1 [TIAB] OR PTEN [TIAB] OR hPTEN [TIAB]) AND ("Urinary Bladder Neoplasms"[Mesh] OR ((Urinary Bladder[tiab] OR Bladder[taib]) AND (cancer*[tiab] OR carcinoma*[tiab] OR neoplasm*[tiab] OR neoplasia*[tiab] OR tumor*[tiab] OR tumour*[tiab]))) |
|  | Breast (Benign) |  | (PTEN protein, human [Supplementary Concept] OR MMAC1 [TIAB] OR mutated in multiple advanced cancers 1 [TIAB] OR phosphatase and tensin homolog (mutated in multiple advanced cancers 1) [TIAB] OR phosphatase and tensin homolog deleted on chromosome 10 [TIAB] OR protein-tyrosine phosphatase PTEN [TIAB] OR PTEN-MMAC1 protein [TIAB] OR PTEN1 [TIAB] OR PTEN_HUMAN [TIAB] OR TEP1 [TIAB] OR TEP1 phosphatase [TIAB] OR Phosphatase And Tensin Homolog [TIAB] OR Phosphatidylinositol 3,4,5-Trisphosphate 3-Phosphatase And Dual-Specificity Protein Phosphatase PTEN [TIAB] OR Phosphatidylinositol-3,4,5-Trisphosphate 3-Phosphatase And Dual-Specificity Protein Phosphatase PTEN [TIAB] OR MMAC1 Phosphatase And Tensin Homolog Deleted On Chromosome 10 [TIAB] OR Mitochondrial Phosphatase And Tensin Protein Alpha [TIAB] OR Phosphatase And Tensin-Like Protein [TIAB] OR Mitochondrial PTENalpha [TIAB] OR EC 3.1.3.16 [TIAB] OR EC 3.1.3.48 [TIAB] OR EC 3.1.3.67 [TIAB] OR 10q23del [TIAB] OR PTENbeta [TIAB] OR GLM2 [TIAB] OR MHAM [TIAB] OR DEC [TIAB] OR BZS [TIAB] OR CWS1 [TIAB] OR PTEN [TIAB] OR hPTEN [TIAB]) AND (Breast[tiab] AND (Cysts[tiab] OR Fibroadenoma[tiab] OR phyllodes[tiab] OR Papillomas[tiab] OR Papillomatosis[tiab] OR Adenoma[tiab]OR Hamartoma[tiab]OR Sclerosing Adenosis[tiab])) |
|  | Cervical Cancer |  | (PTEN protein, human [Supplementary Concept] OR MMAC1 [TIAB] OR mutated in multiple advanced cancers 1 [TIAB] OR phosphatase and tensin homolog (mutated in multiple advanced cancers 1) [TIAB] OR phosphatase and tensin homolog deleted on chromosome 10 [TIAB] OR protein-tyrosine phosphatase PTEN [TIAB] OR PTEN-MMAC1 protein [TIAB] OR PTEN1 [TIAB] OR PTEN_HUMAN [TIAB] OR TEP1 [TIAB] OR TEP1 phosphatase [TIAB] OR Phosphatase And Tensin Homolog [TIAB] OR Phosphatidylinositol 3,4,5-Trisphosphate 3-Phosphatase And Dual-Specificity Protein Phosphatase PTEN [TIAB] OR Phosphatidylinositol-3,4,5-Trisphosphate 3-Phosphatase And Dual-Specificity Protein Phosphatase PTEN [TIAB] OR MMAC1 Phosphatase And Tensin Homolog Deleted On Chromosome 10 [TIAB] OR Mitochondrial Phosphatase And Tensin Protein Alpha [TIAB] OR Phosphatase And Tensin-Like Protein [TIAB] OR Mitochondrial PTENalpha [TIAB] OR EC 3.1.3.16 [TIAB] OR EC 3.1.3.48 [TIAB] OR EC 3.1.3.67 [TIAB] OR 10q23del [TIAB] OR PTENbeta [TIAB] OR GLM2 [TIAB] OR MHAM [TIAB] OR DEC [TIAB] OR BZS [TIAB] OR CWS1 [TIAB] OR PTEN [TIAB] OR hPTEN [TIAB]) AND ("Uterine Cervical Neoplasms"[Mesh] OR ((Cervical[tiab] OR Uterine Cervical[taib]OR Cervix[taib]) AND (cancer*[tiab] OR carcinoma*[tiab] OR neoplasm*[tiab] OR neoplasia*[tiab] OR tumor*[tiab] OR tumour*[tiab]))) |
|  | Ear (Benign) | Hearing Loss | (PTEN protein, human [Supplementary Concept] OR MMAC1 [TIAB] OR mutated in multiple advanced cancers 1 [TIAB] OR phosphatase and tensin homolog (mutated in multiple advanced cancers 1) [TIAB] OR phosphatase and tensin homolog deleted on chromosome 10 [TIAB] OR protein-tyrosine phosphatase PTEN [TIAB] OR PTEN-MMAC1 protein [TIAB] OR PTEN1 [TIAB] OR PTEN_HUMAN [TIAB] OR TEP1 [TIAB] OR TEP1 phosphatase [TIAB] OR Phosphatase And Tensin Homolog [TIAB] OR Phosphatidylinositol 3,4,5-Trisphosphate 3-Phosphatase And Dual-Specificity Protein Phosphatase PTEN [TIAB] OR Phosphatidylinositol-3,4,5-Trisphosphate 3-Phosphatase And Dual-Specificity Protein Phosphatase PTEN [TIAB] OR MMAC1 Phosphatase And Tensin Homolog Deleted On Chromosome 10 [TIAB] OR Mitochondrial Phosphatase And Tensin Protein Alpha [TIAB] OR Phosphatase And Tensin-Like Protein [TIAB] OR Mitochondrial PTENalpha [TIAB] OR EC 3.1.3.16 [TIAB] OR EC 3.1.3.48 [TIAB] OR EC 3.1.3.67 [TIAB] OR 10q23del [TIAB] OR PTENbeta [TIAB] OR GLM2 [TIAB] OR MHAM [TIAB] OR DEC [TIAB] OR BZS [TIAB] OR CWS1 [TIAB] OR PTEN [TIAB] OR hPTEN [TIAB]) AND ("Hearing Loss"[Mesh] OR ((Hearing[tiab] AND Loss[tiab]) OR Hypoacusis[taib]) OR Hearing Impairment[tiab] OR Deafness[tiab] )) |
|  | Eye (Benign) | Cataract | (PTEN protein, human [Supplementary Concept] OR MMAC1 [TIAB] OR mutated in multiple advanced cancers 1 [TIAB] OR phosphatase and tensin homolog (mutated in multiple advanced cancers 1) [TIAB] OR phosphatase and tensin homolog deleted on chromosome 10 [TIAB] OR protein-tyrosine phosphatase PTEN [TIAB] OR PTEN-MMAC1 protein [TIAB] OR PTEN1 [TIAB] OR PTEN_HUMAN [TIAB] OR TEP1 [TIAB] OR TEP1 phosphatase [TIAB] OR Phosphatase And Tensin Homolog [TIAB] OR Phosphatidylinositol 3,4,5-Trisphosphate 3-Phosphatase And Dual-Specificity Protein Phosphatase PTEN [TIAB] OR Phosphatidylinositol-3,4,5-Trisphosphate 3-Phosphatase And Dual-Specificity Protein Phosphatase PTEN [TIAB] OR MMAC1 Phosphatase And Tensin Homolog Deleted On Chromosome 10 [TIAB] OR Mitochondrial Phosphatase And Tensin Protein Alpha [TIAB] OR Phosphatase And Tensin-Like Protein [TIAB] OR Mitochondrial PTENalpha [TIAB] OR EC 3.1.3.16 [TIAB] OR EC 3.1.3.48 [TIAB] OR EC 3.1.3.67 [TIAB] OR 10q23del [TIAB] OR PTENbeta [TIAB] OR GLM2 [TIAB] OR MHAM [TIAB] OR DEC [TIAB] OR BZS [TIAB] OR CWS1 [TIAB] OR PTEN [TIAB] OR hPTEN [TIAB]) AND ("Cataract"[Mesh] OR (Cataracts OR Lens Opacity[taib] OR Pseudoaphakia[tiab] OR (Cataract[tiab] AND Membranous[tiab] ))) |
|  | Gastric Cancer |  | (PTEN protein, human [Supplementary Concept] OR MMAC1 [TIAB] OR mutated in multiple advanced cancers 1 [TIAB] OR phosphatase and tensin homolog (mutated in multiple advanced cancers 1) [TIAB] OR phosphatase and tensin homolog deleted on chromosome 10 [TIAB] OR protein-tyrosine phosphatase PTEN [TIAB] OR PTEN-MMAC1 protein [TIAB] OR PTEN1 [TIAB] OR PTEN_HUMAN [TIAB] OR TEP1 [TIAB] OR TEP1 phosphatase [TIAB] OR Phosphatase And Tensin Homolog [TIAB] OR Phosphatidylinositol 3,4,5-Trisphosphate 3-Phosphatase And Dual-Specificity Protein Phosphatase PTEN [TIAB] OR Phosphatidylinositol-3,4,5-Trisphosphate 3-Phosphatase And Dual-Specificity Protein Phosphatase PTEN [TIAB] OR MMAC1 Phosphatase And Tensin Homolog Deleted On Chromosome 10 [TIAB] OR Mitochondrial Phosphatase And Tensin Protein Alpha [TIAB] OR Phosphatase And Tensin-Like Protein [TIAB] OR Mitochondrial PTENalpha [TIAB] OR EC 3.1.3.16 [TIAB] OR EC 3.1.3.48 [TIAB] OR EC 3.1.3.67 [TIAB] OR 10q23del [TIAB] OR PTENbeta [TIAB] OR GLM2 [TIAB] OR MHAM [TIAB] OR DEC [TIAB] OR BZS [TIAB] OR CWS1 [TIAB] OR PTEN [TIAB] OR hPTEN [TIAB]) AND ((Stomach[tiab] OR Gastric[tiab]) AND (cancer*[tiab] OR carcinoma*[tiab] OR neoplasm*[tiab] OR neoplasia*[tiab] OR tumor*[tiab] OR tumour*[tiab]))) |
|  | Genitourinary (Benign) | Hydrocele | (PTEN protein, human [Supplementary Concept] OR MMAC1 [TIAB] OR mutated in multiple advanced cancers 1 [TIAB] OR phosphatase and tensin homolog (mutated in multiple advanced cancers 1) [TIAB] OR phosphatase and tensin homolog deleted on chromosome 10 [TIAB] OR protein-tyrosine phosphatase PTEN [TIAB] OR PTEN-MMAC1 protein [TIAB] OR PTEN1 [TIAB] OR PTEN_HUMAN [TIAB] OR TEP1 [TIAB] OR TEP1 phosphatase [TIAB] OR Phosphatase And Tensin Homolog [TIAB] OR Phosphatidylinositol 3,4,5-Trisphosphate 3-Phosphatase And Dual-Specificity Protein Phosphatase PTEN [TIAB] OR Phosphatidylinositol-3,4,5-Trisphosphate 3-Phosphatase And Dual-Specificity Protein Phosphatase PTEN [TIAB] OR MMAC1 Phosphatase And Tensin Homolog Deleted On Chromosome 10 [TIAB] OR Mitochondrial Phosphatase And Tensin Protein Alpha [TIAB] OR Phosphatase And Tensin-Like Protein [TIAB] OR Mitochondrial PTENalpha [TIAB] OR EC 3.1.3.16 [TIAB] OR EC 3.1.3.48 [TIAB] OR EC 3.1.3.67 [TIAB] OR 10q23del [TIAB] OR PTENbeta [TIAB] OR GLM2 [TIAB] OR MHAM [TIAB] OR DEC [TIAB] OR BZS [TIAB] OR CWS1 [TIAB] OR PTEN [TIAB] OR hPTEN [TIAB]) AND ("Testicular Hydrocele"[Mesh] OR (Testicular Hydrocele[taib] OR Scrotal Hydrocele[taib] OR Vaginal Hydrocele[tiab] )) |
|  | Head and Neck Cancer | Squamous Cell Carcinoma | (PTEN protein, human [Supplementary Concept] OR MMAC1 [TIAB] OR mutated in multiple advanced cancers 1 [TIAB] OR phosphatase and tensin homolog (mutated in multiple advanced cancers 1) [TIAB] OR phosphatase and tensin homolog deleted on chromosome 10 [TIAB] OR protein-tyrosine phosphatase PTEN [TIAB] OR PTEN-MMAC1 protein [TIAB] OR PTEN1 [TIAB] OR PTEN_HUMAN [TIAB] OR TEP1 [TIAB] OR TEP1 phosphatase [TIAB] OR Phosphatase And Tensin Homolog [TIAB] OR Phosphatidylinositol 3,4,5-Trisphosphate 3-Phosphatase And Dual-Specificity Protein Phosphatase PTEN [TIAB] OR Phosphatidylinositol-3,4,5-Trisphosphate 3-Phosphatase And Dual-Specificity Protein Phosphatase PTEN [TIAB] OR MMAC1 Phosphatase And Tensin Homolog Deleted On Chromosome 10 [TIAB] OR Mitochondrial Phosphatase And Tensin Protein Alpha [TIAB] OR Phosphatase And Tensin-Like Protein [TIAB] OR Mitochondrial PTENalpha [TIAB] OR EC 3.1.3.16 [TIAB] OR EC 3.1.3.48 [TIAB] OR EC 3.1.3.67 [TIAB] OR 10q23del [TIAB] OR PTENbeta [TIAB] OR GLM2 [TIAB] OR MHAM [TIAB] OR DEC [TIAB] OR BZS [TIAB] OR CWS1 [TIAB] OR PTEN [TIAB] OR hPTEN [TIAB]) AND ("Head and Neck Neoplasms"[Mesh] OR ((Head and Neck [taib] OR Upper Aerodigestive Tract [taib]) AND (cancer*[tiab] OR carcinoma*[tiab] OR neoplasm*[tiab] OR neoplasia*[tiab] OR tumor*[tiab] OR tumour*[tiab]))) |
|  | Liver (Benign) | Hepatomegaly | (PTEN protein, human [Supplementary Concept] OR MMAC1 [TIAB] OR mutated in multiple advanced cancers 1 [TIAB] OR phosphatase and tensin homolog (mutated in multiple advanced cancers 1) [TIAB] OR phosphatase and tensin homolog deleted on chromosome 10 [TIAB] OR protein-tyrosine phosphatase PTEN [TIAB] OR PTEN-MMAC1 protein [TIAB] OR PTEN1 [TIAB] OR PTEN_HUMAN [TIAB] OR TEP1 [TIAB] OR TEP1 phosphatase [TIAB] OR Phosphatase And Tensin Homolog [TIAB] OR Phosphatidylinositol 3,4,5-Trisphosphate 3-Phosphatase And Dual-Specificity Protein Phosphatase PTEN [TIAB] OR Phosphatidylinositol-3,4,5-Trisphosphate 3-Phosphatase And Dual-Specificity Protein Phosphatase PTEN [TIAB] OR MMAC1 Phosphatase And Tensin Homolog Deleted On Chromosome 10 [TIAB] OR Mitochondrial Phosphatase And Tensin Protein Alpha [TIAB] OR Phosphatase And Tensin-Like Protein [TIAB] OR Mitochondrial PTENalpha [TIAB] OR EC 3.1.3.16 [TIAB] OR EC 3.1.3.48 [TIAB] OR EC 3.1.3.67 [TIAB] OR 10q23del [TIAB] OR PTENbeta [TIAB] OR GLM2 [TIAB] OR MHAM [TIAB] OR DEC [TIAB] OR BZS [TIAB] OR CWS1 [TIAB] OR PTEN [TIAB] OR hPTEN [TIAB]) AND ("Hepatomegaly"[Mesh] OR (Hepatomegaly[taib] OR Enlarged Liver[taib] )) |
|  | Lung Cancer |  | (PTEN protein, human [Supplementary Concept] OR MMAC1 [TIAB] OR mutated in multiple advanced cancers 1 [TIAB] OR phosphatase and tensin homolog (mutated in multiple advanced cancers 1) [TIAB] OR phosphatase and tensin homolog deleted on chromosome 10 [TIAB] OR protein-tyrosine phosphatase PTEN [TIAB] OR PTEN-MMAC1 protein [TIAB] OR PTEN1 [TIAB] OR PTEN_HUMAN [TIAB] OR TEP1 [TIAB] OR TEP1 phosphatase [TIAB] OR Phosphatase And Tensin Homolog [TIAB] OR Phosphatidylinositol 3,4,5-Trisphosphate 3-Phosphatase And Dual-Specificity Protein Phosphatase PTEN [TIAB] OR Phosphatidylinositol-3,4,5-Trisphosphate 3-Phosphatase And Dual-Specificity Protein Phosphatase PTEN [TIAB] OR MMAC1 Phosphatase And Tensin Homolog Deleted On Chromosome 10 [TIAB] OR Mitochondrial Phosphatase And Tensin Protein Alpha [TIAB] OR Phosphatase And Tensin-Like Protein [TIAB] OR Mitochondrial PTENalpha [TIAB] OR EC 3.1.3.16 [TIAB] OR EC 3.1.3.48 [TIAB] OR EC 3.1.3.67 [TIAB] OR 10q23del [TIAB] OR PTENbeta [TIAB] OR GLM2 [TIAB] OR MHAM [TIAB] OR DEC [TIAB] OR BZS [TIAB] OR CWS1 [TIAB] OR PTEN [TIAB] OR hPTEN [TIAB]) AND ("Lung Neoplasms"[Mesh] OR ((Lung[tiab] OR Pulmonary[tiab]) AND (cancer*[tiab] OR carcinoma*[tiab] OR neoplasm*[tiab] OR neoplasia*[tiab] OR tumor*[tiab] OR tumour*[tiab]))) |
|  | Ovarian Cancer |  | (PTEN protein, human [Supplementary Concept] OR MMAC1 [TIAB] OR mutated in multiple advanced cancers 1 [TIAB] OR phosphatase and tensin homolog (mutated in multiple advanced cancers 1) [TIAB] OR phosphatase and tensin homolog deleted on chromosome 10 [TIAB] OR protein-tyrosine phosphatase PTEN [TIAB] OR PTEN-MMAC1 protein [TIAB] OR PTEN1 [TIAB] OR PTEN_HUMAN [TIAB] OR TEP1 [TIAB] OR TEP1 phosphatase [TIAB] OR Phosphatase And Tensin Homolog [TIAB] OR Phosphatidylinositol 3,4,5-Trisphosphate 3-Phosphatase And Dual-Specificity Protein Phosphatase PTEN [TIAB] OR Phosphatidylinositol-3,4,5-Trisphosphate 3-Phosphatase And Dual-Specificity Protein Phosphatase PTEN [TIAB] OR MMAC1 Phosphatase And Tensin Homolog Deleted On Chromosome 10 [TIAB] OR Mitochondrial Phosphatase And Tensin Protein Alpha [TIAB] OR Phosphatase And Tensin-Like Protein [TIAB] OR Mitochondrial PTENalpha [TIAB] OR EC 3.1.3.16 [TIAB] OR EC 3.1.3.48 [TIAB] OR EC 3.1.3.67 [TIAB] OR 10q23del [TIAB] OR PTENbeta [TIAB] OR GLM2 [TIAB] OR MHAM [TIAB] OR DEC [TIAB] OR BZS [TIAB] OR CWS1 [TIAB] OR PTEN [TIAB] OR hPTEN [TIAB]) AND ("Ovarian Neoplasms"[Mesh] OR ((ovary[tiab] OR ovaries[tiab] OR ovarian[taib]) AND (cancer*[tiab] OR carcinoma*[tiab] OR neoplasm*[tiab] OR neoplasia*[tiab] OR tumor*[tiab] OR tumour*[tiab]))) |
|  | Skin Cancer (Non-Melanoma) | Squamous Cell Carcinoma | (PTEN protein, human [Supplementary Concept] OR MMAC1 [TIAB] OR mutated in multiple advanced cancers 1 [TIAB] OR phosphatase and tensin homolog (mutated in multiple advanced cancers 1) [TIAB] OR phosphatase and tensin homolog deleted on chromosome 10 [TIAB] OR protein-tyrosine phosphatase PTEN [TIAB] OR PTEN-MMAC1 protein [TIAB] OR PTEN1 [TIAB] OR PTEN_HUMAN [TIAB] OR TEP1 [TIAB] OR TEP1 phosphatase [TIAB] OR Phosphatase And Tensin Homolog [TIAB] OR Phosphatidylinositol 3,4,5-Trisphosphate 3-Phosphatase And Dual-Specificity Protein Phosphatase PTEN [TIAB] OR Phosphatidylinositol-3,4,5-Trisphosphate 3-Phosphatase And Dual-Specificity Protein Phosphatase PTEN [TIAB] OR MMAC1 Phosphatase And Tensin Homolog Deleted On Chromosome 10 [TIAB] OR Mitochondrial Phosphatase And Tensin Protein Alpha [TIAB] OR Phosphatase And Tensin-Like Protein [TIAB] OR Mitochondrial PTENalpha [TIAB] OR EC 3.1.3.16 [TIAB] OR EC 3.1.3.48 [TIAB] OR EC 3.1.3.67 [TIAB] OR 10q23del [TIAB] OR PTENbeta [TIAB] OR GLM2 [TIAB] OR MHAM [TIAB] OR DEC [TIAB] OR BZS [TIAB] OR CWS1 [TIAB] OR PTEN [TIAB] OR hPTEN [TIAB]) AND ("Skin Neoplasms"[Mesh] OR (Skin[taib] AND (cancer*[tiab] OR carcinoma*[tiab] OR neoplasm*[tiab] OR neoplasia*[tiab] OR tumor*[tiab] OR tumour*[tiab]))) |
|  | Spleen (Benign) | Splenomegaly | (PTEN protein, human [Supplementary Concept] OR MMAC1 [TIAB] OR mutated in multiple advanced cancers 1 [TIAB] OR phosphatase and tensin homolog (mutated in multiple advanced cancers 1) [TIAB] OR phosphatase and tensin homolog deleted on chromosome 10 [TIAB] OR protein-tyrosine phosphatase PTEN [TIAB] OR PTEN-MMAC1 protein [TIAB] OR PTEN1 [TIAB] OR PTEN_HUMAN [TIAB] OR TEP1 [TIAB] OR TEP1 phosphatase [TIAB] OR Phosphatase And Tensin Homolog [TIAB] OR Phosphatidylinositol 3,4,5-Trisphosphate 3-Phosphatase And Dual-Specificity Protein Phosphatase PTEN [TIAB] OR Phosphatidylinositol-3,4,5-Trisphosphate 3-Phosphatase And Dual-Specificity Protein Phosphatase PTEN [TIAB] OR MMAC1 Phosphatase And Tensin Homolog Deleted On Chromosome 10 [TIAB] OR Mitochondrial Phosphatase And Tensin Protein Alpha [TIAB] OR Phosphatase And Tensin-Like Protein [TIAB] OR Mitochondrial PTENalpha [TIAB] OR EC 3.1.3.16 [TIAB] OR EC 3.1.3.48 [TIAB] OR EC 3.1.3.67 [TIAB] OR 10q23del [TIAB] OR PTENbeta [TIAB] OR GLM2 [TIAB] OR MHAM [TIAB] OR DEC [TIAB] OR BZS [TIAB] OR CWS1 [TIAB] OR PTEN [TIAB] OR hPTEN [TIAB]) AND ("Splenomegaly"[Mesh] OR (Splenomegaly[taib] OR Enlarged Spleen[taib] )) |
| *RECQL* | Ovarian Cancer |  | (RecQ Helicases [MESH] OR RECQL protein, human [Supplementary Concept] OR RECQL [TIAB]) AND ("Ovarian Neoplasms"[Mesh] OR ((ovary[tiab] OR ovaries[tiab] OR ovarian[taib]) AND (cancer*[tiab] OR carcinoma*[tiab] OR neoplasm*[tiab] OR neoplasia*[tiab] OR tumor*[tiab] OR tumour*[tiab]))) |
| *STK11* | Nose (Benign) | Nasal Polyps | (Peutz-Jeghers Syndrome [MESH] OR STK11 protein, human [Supplementary Concept] OR LKB1 [TIAB] OR PJS [TIAB] OR STK11_HUMAN [TIAB] OR serine/threonine kinase 11 (Peutz-Jeghers syndrome) [TIAB] OR Serine/threonine-protein kinase 11 [TIAB] OR Serine/Threonine Kinase 11 [TIAB] OR Polarization-Related Protein LKB1 [TIAB] OR Renal Carcinoma Antigen NY-REN-19 [TIAB] OR Liver Kinase B1 [TIAB] OR EC 2.7.11.1 [TIAB] OR HLKB1 [TIAB] OR Serine/Threonine-Protein Kinase STK11 [TIAB] OR Serine/Threonine-Protein Kinase LKB11 [TIAB] OR STK11 [TIAB] OR hSTK11 [TIAB]) AND ("Nasal Polyp" [TIAB] OR "Nasal Polyps" [Mesh]) |
|  | Ovarian Cancer |  | (Peutz-Jeghers Syndrome [MESH] OR STK11 protein, human [Supplementary Concept] OR LKB1 [TIAB] OR PJS [TIAB] OR STK11_HUMAN [TIAB] OR serine/threonine kinase 11 (Peutz-Jeghers syndrome) [TIAB] OR Serine/threonine-protein kinase 11 [TIAB] OR Serine/Threonine Kinase 11 [TIAB] OR Polarization-Related Protein LKB1 [TIAB] OR Renal Carcinoma Antigen NY-REN-19 [TIAB] OR Liver Kinase B1 [TIAB] OR EC 2.7.11.1 [TIAB] OR HLKB1 [TIAB] OR Serine/Threonine-Protein Kinase STK11 [TIAB] OR Serine/Threonine-Protein Kinase LKB11 [TIAB] OR STK11 [TIAB] OR hSTK11 [TIAB]) AND ("Ovarian Neoplasms"[Mesh] OR ((ovary[tiab] OR ovaries[tiab] OR ovarian[taib]) AND (cancer*[tiab] OR carcinoma*[tiab] OR neoplasm*[tiab] OR neoplasia*[tiab] OR tumor*[tiab] OR tumour*[tiab]))) |
|  | Thyroid Cancer |  | (Peutz-Jeghers Syndrome [MESH] OR STK11 protein, human [Supplementary Concept] OR LKB1 [TIAB] OR PJS [TIAB] OR STK11_HUMAN [TIAB] OR serine/threonine kinase 11 (Peutz-Jeghers syndrome) [TIAB] OR Serine/threonine-protein kinase 11 [TIAB] OR Serine/Threonine Kinase 11 [TIAB] OR Polarization-Related Protein LKB1 [TIAB] OR Renal Carcinoma Antigen NY-REN-19 [TIAB] OR Liver Kinase B1 [TIAB] OR EC 2.7.11.1 [TIAB] OR HLKB1 [TIAB] OR Serine/Threonine-Protein Kinase STK11 [TIAB] OR Serine/Threonine-Protein Kinase LKB11 [TIAB] OR STK11 [TIAB] OR hSTK11 [TIAB]) AND ("Thyroid Neoplasms"[Mesh] OR (Thyroid[tiab] AND (cancer*[tiab] OR carcinoma*[tiab] OR neoplasm*[tiab] OR neoplasia*[tiab] OR tumor*[tiab] OR tumour*[tiab]))) |
| *TP53* | Gastric Cancer |  | (Genes, p53 [MESH] OR cellular tumor antigen p53 [TIAB] OR P53 tumor suppressor [TIAB] OR P53_HUMAN [TIAB] OR phosphoprotein p53 [TIAB] OR transformation-related protein 53 [TIAB] OR TRP53 [TIAB] OR tumor protein p53 (Li-Fraumeni syndrome) [TIAB] OR tumor suppressor p53 [TIAB] OR Tumor Protein P53 [TIAB] OR Antigen NY-CO-13 [TIAB] OR Mutant Tumor Protein 53 [TIAB] OR Li-Fraumeni Syndrome [TIAB] OR Tumor Supressor P53 [TIAB] OR Tumor Protein 53 [TIAB] OR BCC7 [TIAB] OR LFS1 [TIAB] OR TP53 [TIAB] OR P53 [TIAB]) AND ((Stomach[tiab] OR Gastric[tiab]) AND (cancer*[tiab] OR carcinoma*[tiab] OR neoplasm*[tiab] OR neoplasia*[tiab] OR tumor*[tiab] OR tumour*[tiab]))) |
|  | Head and Neck Cancer |  | (Genes, p53 [MESH] OR cellular tumor antigen p53 [TIAB] OR P53 tumor suppressor [TIAB] OR P53_HUMAN [TIAB] OR phosphoprotein p53 [TIAB] OR transformation-related protein 53 [TIAB] OR TRP53 [TIAB] OR tumor protein p53 (Li-Fraumeni syndrome) [TIAB] OR tumor suppressor p53 [TIAB] OR Tumor Protein P53 [TIAB] OR Antigen NY-CO-13 [TIAB] OR Mutant Tumor Protein 53 [TIAB] OR Li-Fraumeni Syndrome [TIAB] OR Tumor Supressor P53 [TIAB] OR Tumor Protein 53 [TIAB] OR BCC7 [TIAB] OR LFS1 [TIAB] OR TP53 [TIAB] OR P53 [TIAB]) AND ("Head and Neck Neoplasms"[Mesh] OR ((Head and Neck [taib] OR Upper Aerodigestive Tract [taib]) AND (cancer*[tiab] OR carcinoma*[tiab] OR neoplasm*[tiab] OR neoplasia*[tiab] OR tumor*[tiab] OR tumour*[tiab]))) |
|  | Lung Cancer |  | (Genes, p53 [MESH] OR cellular tumor antigen p53 [TIAB] OR P53 tumor suppressor [TIAB] OR P53_HUMAN [TIAB] OR phosphoprotein p53 [TIAB] OR transformation-related protein 53 [TIAB] OR TRP53 [TIAB] OR tumor protein p53 (Li-Fraumeni syndrome) [TIAB] OR tumor suppressor p53 [TIAB] OR Tumor Protein P53 [TIAB] OR Antigen NY-CO-13 [TIAB] OR Mutant Tumor Protein 53 [TIAB] OR Li-Fraumeni Syndrome [TIAB] OR Tumor Supressor P53 [TIAB] OR Tumor Protein 53 [TIAB] OR BCC7 [TIAB] OR LFS1 [TIAB] OR TP53 [TIAB] OR P53 [TIAB]) AND ("Lung Neoplasms"[Mesh] OR ((Lung[tiab] OR Pulmonary[tiab]) AND (cancer*[tiab] OR carcinoma*[tiab] OR neoplasm*[tiab] OR neoplasia*[tiab] OR tumor*[tiab] OR tumour*[tiab]))) |
|  | Melanoma |  | (Genes, p53 [MESH] OR cellular tumor antigen p53 [TIAB] OR P53 tumor suppressor [TIAB] OR P53_HUMAN [TIAB] OR phosphoprotein p53 [TIAB] OR transformation-related protein 53 [TIAB] OR TRP53 [TIAB] OR tumor protein p53 (Li-Fraumeni syndrome) [TIAB] OR tumor suppressor p53 [TIAB] OR Tumor Protein P53 [TIAB] OR Antigen NY-CO-13 [TIAB] OR Mutant Tumor Protein 53 [TIAB] OR Li-Fraumeni Syndrome [TIAB] OR Tumor Supressor P53 [TIAB] OR Tumor Protein 53 [TIAB] OR BCC7 [TIAB] OR LFS1 [TIAB] OR TP53 [TIAB] OR P53 [TIAB]) AND ("Melanoma" [TIAB] OR " Melanoma" [Mesh]) |
|  | Nasopharyngeal Cancer |  | (Genes, p53 [MESH] OR cellular tumor antigen p53 [TIAB] OR P53 tumor suppressor [TIAB] OR P53_HUMAN [TIAB] OR phosphoprotein p53 [TIAB] OR transformation-related protein 53 [TIAB] OR TRP53 [TIAB] OR tumor protein p53 (Li-Fraumeni syndrome) [TIAB] OR tumor suppressor p53 [TIAB] OR Tumor Protein P53 [TIAB] OR Antigen NY-CO-13 [TIAB] OR Mutant Tumor Protein 53 [TIAB] OR Li-Fraumeni Syndrome [TIAB] OR Tumor Supressor P53 [TIAB] OR Tumor Protein 53 [TIAB] OR BCC7 [TIAB] OR LFS1 [TIAB] OR TP53 [TIAB] OR P53 [TIAB]) AND ("Nasopharyngeal Carcinoma"[Mesh] OR ((Nasopharyngeal[tiab]) AND (cancer*[tiab] OR carcinoma*[tiab] OR neoplasm*[tiab] OR neoplasia*[tiab] OR tumor*[tiab] OR tumour*[tiab]))) |
|  | Ovarian Cancer |  | (Genes, p53 [MESH] OR cellular tumor antigen p53 [TIAB] OR P53 tumor suppressor [TIAB] OR P53_HUMAN [TIAB] OR phosphoprotein p53 [TIAB] OR transformation-related protein 53 [TIAB] OR TRP53 [TIAB] OR tumor protein p53 (Li-Fraumeni syndrome) [TIAB] OR tumor suppressor p53 [TIAB] OR Tumor Protein P53 [TIAB] OR Antigen NY-CO-13 [TIAB] OR Mutant Tumor Protein 53 [TIAB] OR Li-Fraumeni Syndrome [TIAB] OR Tumor Supressor P53 [TIAB] OR Tumor Protein 53 [TIAB] OR BCC7 [TIAB] OR LFS1 [TIAB] OR TP53 [TIAB] OR P53 [TIAB]) AND ("Ovarian Neoplasms"[Mesh] OR ((ovary[tiab] OR ovaries[tiab] OR ovarian[taib]) AND (cancer*[tiab] OR carcinoma*[tiab] OR neoplasm*[tiab] OR neoplasia*[tiab] OR tumor*[tiab] OR tumour*[tiab]))) |
|  | Prostate Cancer |  | (Genes, p53 [MESH] OR cellular tumor antigen p53 [TIAB] OR P53 tumor suppressor [TIAB] OR P53_HUMAN [TIAB] OR phosphoprotein p53 [TIAB] OR transformation-related protein 53 [TIAB] OR TRP53 [TIAB] OR tumor protein p53 (Li-Fraumeni syndrome) [TIAB] OR tumor suppressor p53 [TIAB] OR Tumor Protein P53 [TIAB] OR Antigen NY-CO-13 [TIAB] OR Mutant Tumor Protein 53 [TIAB] OR Li-Fraumeni Syndrome [TIAB] OR Tumor Supressor P53 [TIAB] OR Tumor Protein 53 [TIAB] OR BCC7 [TIAB] OR LFS1 [TIAB] OR TP53 [TIAB] OR P53 [TIAB]) AND ("Prostatic Neoplasms"[Mesh] OR ((Prostate [tiab] OR Prostatic[tiab]) AND (cancer*[tiab] OR carcinoma*[tiab] OR neoplasm*[tiab] OR neoplasia*[tiab] OR tumor*[tiab] OR tumour*[tiab]))) |
|  | Skin Cancer (Non-Melanoma) | Basal Cell Carcinoma | (Genes, p53 [MESH] OR cellular tumor antigen p53 [TIAB] OR P53 tumor suppressor [TIAB] OR P53_HUMAN [TIAB] OR phosphoprotein p53 [TIAB] OR transformation-related protein 53 [TIAB] OR TRP53 [TIAB] OR tumor protein p53 (Li-Fraumeni syndrome) [TIAB] OR tumor suppressor p53 [TIAB] OR Tumor Protein P53 [TIAB] OR Antigen NY-CO-13 [TIAB] OR Mutant Tumor Protein 53 [TIAB] OR Li-Fraumeni Syndrome [TIAB] OR Tumor Supressor P53 [TIAB] OR Tumor Protein 53 [TIAB] OR BCC7 [TIAB] OR LFS1 [TIAB] OR TP53 [TIAB] OR P53 [TIAB]) AND ("Skin Neoplasms"[Mesh] OR (Skin[taib] AND (cancer*[tiab] OR carcinoma*[tiab] OR neoplasm*[tiab] OR neoplasia*[tiab] OR tumor*[tiab] OR tumour*[tiab]))) |
|  | Wilms Tumor |  | (Genes, p53 [MESH] OR cellular tumor antigen p53 [TIAB] OR P53 tumor suppressor [TIAB] OR P53_HUMAN [TIAB] OR phosphoprotein p53 [TIAB] OR transformation-related protein 53 [TIAB] OR TRP53 [TIAB] OR tumor protein p53 (Li-Fraumeni syndrome) [TIAB] OR tumor suppressor p53 [TIAB] OR Tumor Protein P53 [TIAB] OR Antigen NY-CO-13 [TIAB] OR Mutant Tumor Protein 53 [TIAB] OR Li-Fraumeni Syndrome [TIAB] OR Tumor Supressor P53 [TIAB] OR Tumor Protein 53 [TIAB] OR BCC7 [TIAB] OR LFS1 [TIAB] OR TP53 [TIAB] OR P53 [TIAB]) AND ("Wilms Tumor"[Mesh] OR (malignant kidney tumor [tiab] OR Wilms Tumor[tiab] OR Wilms' Tumor[taib])) |
