# Supplementary material for: Disease Spectrum of Breast Cancer Susceptibility Genes
Source: Front Oncol. 2021 Apr 20;11:663419. doi: 10.3389/fonc.2021.663419 (PMC8093501; doi:10.3389/fonc.2021.663419)
Supplement: Supplementary file 3 [file Table_3.docx]

**Supplementary Table 3. Diseases associated with breast cancer susceptibility genes**

| **Disease** | **Breast Cancer Susceptibility Genes** | | | | | | | | | | | |
| --- | --- | --- | --- | --- | --- | --- | --- | --- | --- | --- | --- | --- |
|  | *ATM* | *BARD1* | *BRCA1* | *BRCA2* | *CDH1* | *CHEK2* | *NF1* | *PALB2* | *PTEN* | *RECQL* | *STK11* | *TP53* |
| Adrenocortical Carcinoma |  |  |  |  |  |  |  |  |  |  |  | 1 |
| Autism |  |  |  |  |  |  |  |  | 1 |  |  |  |
| BCD Syndrome* |  |  |  |  | 1 |  |  |  |  |  |  |  |
| Bone Dysplasia |  |  |  |  |  |  | 1 |  |  |  |  |  |
| Brain Tumor |  |  |  |  |  |  | 1 |  | 1 |  |  | 1 |
| Breast Cancer | 1 | 1 | 1 | 1 | 1 | 1 | 1 | 1 | 1 | 1 | 1 | 1 |
| Cafe-Au-Lait Spots |  |  |  |  |  |  | 1 |  |  |  |  |  |
| Cerebrovascular Malformation |  |  |  |  |  |  |  |  | 1 |  |  |  |
| Cervical Cancer |  |  |  |  |  |  |  |  |  |  | 1 |  |
| Colorectal Cancer | 1 |  |  |  |  | 1 |  |  | 1 |  | 1 | 1 |
| Endometrial Cancer |  |  |  |  |  |  |  |  | 1 |  | 1 |  |
| Facial Papules |  |  |  |  |  |  |  |  | 1 |  |  |  |
| Gastric Cancer | 1 |  |  |  | 1 | 1 |  | 1 |  |  | 1 |  |
| GI Hamartomatous Polyps |  |  |  |  |  |  |  |  | 1 |  | 1 |  |
| GIST |  |  |  |  |  |  | 1 |  |  |  |  |  |
| Hepatobiliary Cancer |  |  |  |  |  |  |  |  |  |  | 1 | 1 |
| Intellectual Disability |  |  |  |  |  |  | 1 |  |  |  |  |  |
| Iris Hamartoma |  |  |  |  |  |  | 1 |  |  |  |  |  |
| Kidney Cancer |  |  |  |  |  | 1 |  |  | 1 |  |  |  |
| Leukemia |  |  |  |  |  |  | 1 |  |  |  |  |  |
| Lipoma |  |  |  |  |  |  |  |  | 1 |  |  |  |
| Lung Cancer |  |  |  |  |  |  |  |  |  |  | 1 |  |
| Macrocephaly |  |  |  |  |  |  |  |  | 1 |  |  |  |
| Melanoma |  |  |  | 1 |  |  |  |  | 1 |  |  |  |
| Neurofibroma |  |  |  |  |  |  | 1 |  |  |  |  |  |
| Non-Epithelial Ovarian Tumor |  |  |  |  |  |  |  |  |  |  | 1 |  |
| Oral Mucosal Papillomatosis |  |  |  |  |  |  |  |  | 1 |  |  |  |
| Ovarian Cancer |  |  | 1 | 1 |  |  |  | 1 |  |  |  |  |
| Ovarian SCST |  |  |  |  |  |  |  |  |  |  | 1 |  |
| Osteosarcoma |  |  |  |  |  |  |  |  |  |  |  | 1 |
| Pancreatic Cancer | 1 |  | 1 | 1 |  |  |  | 1 |  |  | 1 | 1 |
| Paraganglioma |  |  |  |  |  |  | 1 |  |  |  |  |  |
| Pheochromocytoma |  |  |  |  |  |  | 1 |  |  |  |  |  |
| Prostate Cancer | 1 |  | 1 | 1 |  | 1 |  | 1 |  |  |  |  |
| Pulmonary Stenosis |  |  |  |  |  |  | 1 |  |  |  |  |  |
| Sarcoma |  |  |  |  |  | 1 | 1 |  |  |  |  | 1 |
| Skin (Benign)** |  |  |  |  |  |  | 1 |  | 1 |  | 1 |  |
| Small Intestine Cancer |  |  |  |  |  |  |  |  |  |  | 1 |  |
| Testicular SCST |  |  |  |  |  |  |  |  |  |  | 1 |  |
| Thyroid (Benign)** |  |  |  |  |  |  |  |  | 1 |  |  |  |
| Thyroid Cancer |  |  |  |  |  | 1 |  |  | 1 |  |  |  |
| Uterine Fibroid |  |  |  |  |  |  |  |  | 1 |  |  |  |

Abbreviations: GI, gastrointestinal; BCD, blepharocheilodontic; SCST, sex cord-stromal tumor; GIST, gastrointestinal stromal tumor

Note: The number ‘1’ indicates that the gene was associated with the disease/cancer in the resource. Blank space indicates association was not found in the resource.

* BCD syndrome consists of: Facial Dysmorphism, Hypertelorism, Imperforate anus, Distichiasis, Clinodactyly, Hypoplastic nails, Choanal atresia, Cleft palate, benign teeth disorder.

**Benign: This may mean a verity of benign diseases related to the organ and have been elaborated in Supplement Table 1
